# A unified fixed point approach to study the existence of solutions for a class of fractional boundary value problems arising in a chemical graph theory

**DOI:** 10.1371/journal.pone.0270148

**Published:** 2022-08-12

**Authors:** Wutiphol Sintunavarat, Ali Turab

**Affiliations:** Department of Mathematics and Statistics, Faculty of Science and Technology, Thammasat University Rangsit Center, Pathum Thani, Thailand; Hodeidah University, YEMEN

## Abstract

A theory of chemical graphs is a part of mathematical chemistry concerned with the effects of connectedness in chemical graphs. Several researchers have studied the solutions of fractional differential equations using the concept of star graphs. They employed star graphs because their technique requires a central node with links to adjacent vertices but no edges between nodes. The purpose of this paper is to extend the method’s range by introducing the concept of an octane graph, which is an essential organic compound having the formula *C*_8_*H*_18_. In this manner, we analyze a graph with vertices annotated by 0 or 1, which is influenced by the structure of the chemical substance octane, and formulate a fractional boundary value problem on each of the graph’s edges. We use the Schaefer and Krasnoselskii fixed point theorems to investigate the existence of solutions to the presented boundary value problems in the framework of the Caputo fractional derivative. Finally, two examples are provided to highlight the importance of our results in this area of study.

## 1 Introduction

Chemical graph theory is concerned with all elements of graph theory’s application to chemistry. In contrast to graph theory, the term chemical emphasizes that one may rely on the intuitive understanding of several concepts and theorems in chemical graph theory rather than precise mathematical proofs. On the other hand, graph theory is used to mathematically portray the structural properties of chemical compounds to understand them. A substance’s physical properties, such as its boiling point, are related to its geometric structure.

The concept of chemical indices is one of the most fundamental ideas in chemical graph theory. This is done by associating a numerical value with a graph structure that frequently has some relationship with the characteristics of the relevant molecules. As a result, these chemical indices are often presented as identifiers of chemical components. From a graph-theoretical standpoint, investigating such a chemical index often entails researching its behavior in various graphs, particularly minima and maxima, as well as upper and lower limits in terms of various graph characteristics.

Graph theory is closely connected to topology (in fact, it is one-dimensional topology [1]), probability, group theory, matrix theory, set theory, numerical analysis, and combinatorics. It has been used in a wide range of subjects, including psychology [2] and nuclear physics [3], economics [4] and theoretical physics [5], biomathematics [6] and linguistics [7], technology [8] and anthropology [9], sociology [10] and zoology [11], biology [12] and engineering [13], computer science [14] and geography [15], and so on.

Chemical graph theory has grown significantly in popularity in recent years (for the detail, see [16–18]). Numerous factors contribute to graph theory’s growing prominence in chemistry (see [19–21]). First, few concepts in the natural sciences are more closely related to the concept of a graph than the institutional formula of a chemical compound (see [22]). Thus, it would seem that (chemical) graph theory provides the natural language of chemistry through which scientists interact. Second, graph theory enables researchers to make many intuitive assumptions about the composition and reactivity of diverse substances using simple principles. Thirdly, graph theory may describe, classify, and categorize a vast range of chemical interactions (for the detail, see [23–25]). Lastly, graphs provide practical tools for the computer-assisted synthesis design (see [26, 27]).

In [28], Lumer modified the specified local operators on ramification spaces and investigated the solutions of evolution equations on graphs. After that, some researchers examined the solutions of differential equations on graphs by using different methods (for the detail, see [29, 30]).

**Fig 1:**
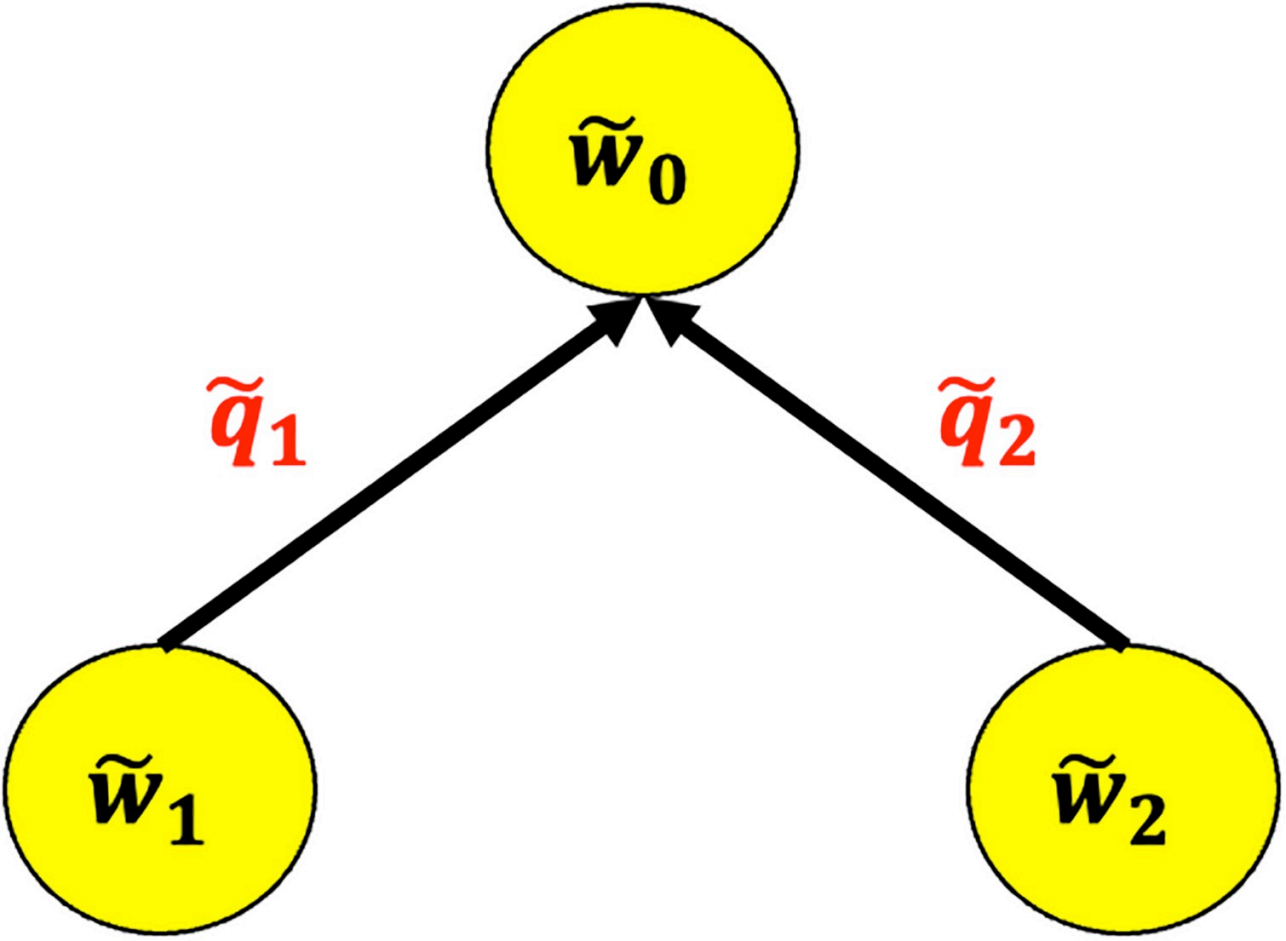
A structure of a star graph $G^{⋆}$ having one junction node and two edges.

However, there are just a few research on boundary value problems with graphs in which particular fixed point methods have shown the existence of solutions (see [31, 32]). In such studies, the authors utilized the concept of a star graph, which has only one junction node (see Fig 1). Since then, various authors have used notable methods to extend the problem in different directions see [33–38] and the references within.

**Fig 2:**
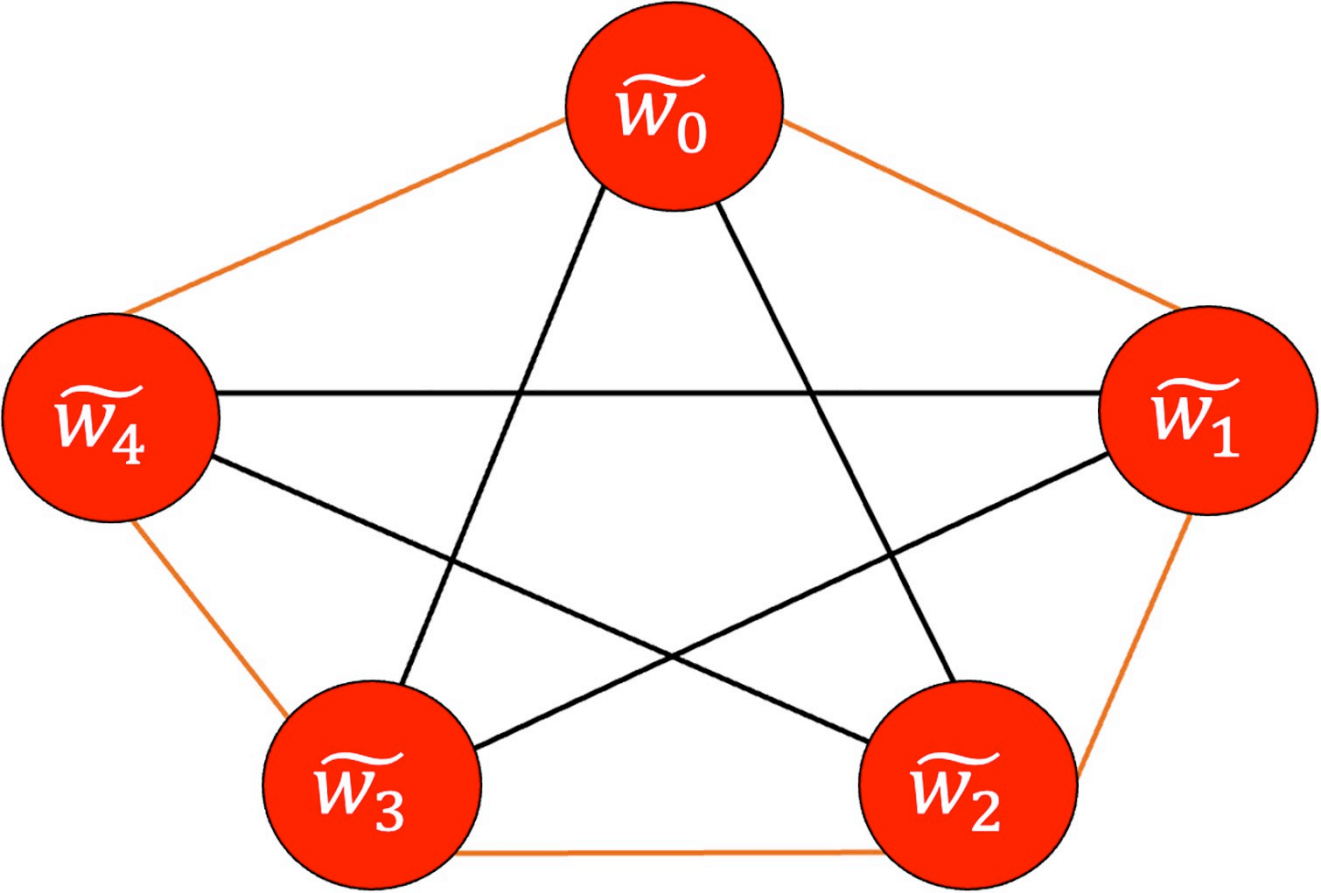
An example of a non-planar graph.

**Fig 3:**
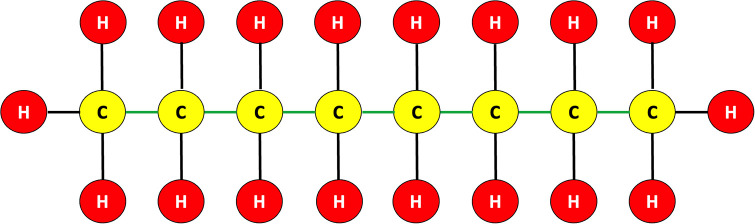
Chemical bonds of an octane compound *C*_8_*H*_18_ having more than one junction nodes.

The methods described in [31, 32] for determining the origin at edges other than the junction node ${\tilde{w}}_{0}$ are inadequate since graphs might contain several junction nodes in general (for examples, see Figs 2 and 3).

**Fig 4:**
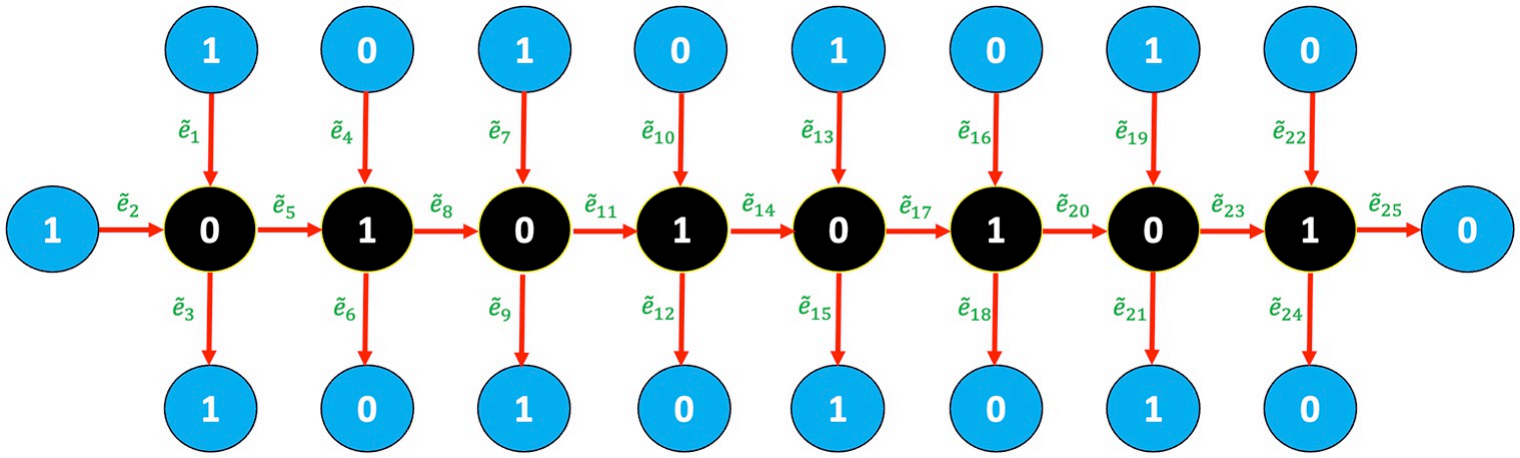
A structure of an octane compound *C*_8_*H*_18_ with labeled vertices 0 or 1.

Additionally, the authors of [31, 32] treated the length of each edge as a variable, but the length of all edges may be considered constant from the start. Here, we use a novel approach in which we assign a value of 0 or 1 to the vertices of the proposed graph with $|{\tilde{e}}_{k}|=1,$ for all *k* = 1, 2, …, 25 (see Fig 4).

By utilizing the ideas mentioned above, here, we investigate the existence of solutions to the boundary value problem, which is stated for each *k* = 1, 2, …, 25 by

$$\begin{array}{c} \left\{ \begin{array}{cc} D^{p}y_{k}\left(s\right) & =Z_{k}\left(s,y_{k}\left(s\right),D^{q}y_{k}\left(s\right),y_{k}^{\prime }\left(s\right),y_{k}^{\prime \prime }\left(s\right)\right)\;\left(s\in \left[0,1\right]\right),\\ \mu_{1}y_{k}\left(0\right) & +\mu_{2}(D^{q}y_{k}\left(0\right))=\mu_{3}\int_{0}^{1}y_{k}\left(\varsigma \right)d\varsigma ,\\ \mu_{1}y_{k}\left(1\right) & +\mu_{2}(D^{q}y_{k}\left(1\right))=\mu_{3}\int_{0}^{1}y_{k}\left(\varsigma \right)d\varsigma , \end{array}\right. \end{array}$$ (1.1)

where $y_{k}:\left[0,1\right]\to R$ is an unknown function, $\mu_{1},\mu_{2},\mu_{3}\in R\backslash \left\{0\right\}$ with *μ*_3_ ≠ *μ*_1_, $D^{p}$ and $D^{q}$ represent the Caputo fractional derivative of orders *p* ∈ (1, 2] and *q* ∈ (0, 1), respectively. Also, $Z_{k}:\left[0,1\right]\times R\times R\times R\times R\to R$ is a continuously differentiable function for *k* = 1, 2, …, 25.

In this way, the orientation of the linked edge determines the label given to each vertex. When we proceed along a random edge, the starting and ending vertex labels are interpreted as 0 and 1, and vice versa. As a consequence, some vertices may have the labels 0 and 1, and the origin of each edge is not constant; it fluctuates depending on the path of motion along the border. We are not obliged to normalize the length of each edge using the provided adjustment, and we may also pick one of the associated edge’s two vertices as the origin using such procedures.

There are two points on each edge where unknown functions’ boundary values and their *q*−derivatives are linearly combined. This study shows that the anonymous functions’ integral is a multiple of these combinations. Additionally, it is worth noting that the solutions derived for the proposed boundary value problem (1.1) can be applied in various chemical graph theory applications. As a result, we assert that this generic concept may be beneficial to future work by young scholars.

On the other hand, numerous advanced fractional modeling techniques are discussed in the literature, notably (but not limited to) the well-known Caputo and Riemann–Liouville operators (for the detail, see [39–45]). This decade has seen the introduction of several novel modifications of the Hadamard, Caputo–Hadamard, and Hilfer operators and numerous simulation efforts using these new operators (for the detail, see [46–50]). Fabrizio and Caputo suggested a new formulation of a fractional framework without singularity six years ago (see [51]). Shortly after this work, Nieto and Losada concentrated on significant computational aspects (see [52]). The inclusion of nonsingular operators resulted in many research publications on fractional modeling (for example, see [53–55]).

This study aims to establish the existence of solutions to the specified boundary value problem (1.1) by using well-known fixed point techniques. Finally, two examples are presented to emphasize the significance of our results in this field of study.

## 2 Preliminaries

The succeeding results will be needed in the following sections.

**Definition 2.1** ([51]). Let *p* > 0. The Caputo fractional derivative of order *p* for a function $Z\in C^{\chi }([a,b],R)$ is defined by

$$\begin{array}{c} D^{p}Z\left(s\right)=\frac{1}{\Gamma (\chi -p)}\int_{0}^{s}{\left(s-\varsigma \right)}^{\chi -p-1}Z^{\left(\chi \right)}\left(\varsigma \right)d\varsigma \;\left(\chi -1<p<\chi ,\;\chi =\left[p\right]+1\right). \end{array}$$

For *p* > 0, the general solution of $D^{p}y\left(s\right)=0$ is given as

$$\begin{array}{c} y\left(s\right)=z_{0}+z_{1}s+z_{2}s^{2}+\cdots +z_{n-1}s^{n-1}, \end{array}$$

where $z_{k}\in R,$ *k* = 0, 1, …, *n* − 1 (*n* − 1 < *p* < *n*, *n* = [*p*] + 1).

**Lemma 2.2**. *Suppose that* ψ∈C([0,1],R). *Then* $y^{⋆}:\left[0,1\right]\to R$ *is a solution of*

$$\begin{array}{c} \left\{ \begin{array}{cc} D^{p}y\left(s\right) & =\psi (t)\;(s\in [0,1]),\\ \mu_{1}y\left(0\right) & +\mu_{2}(D^{q}y\left(0\right))=\mu_{3}\int_{0}^{1}y\left(\varsigma \right)d\varsigma ,\\ \mu_{1}y\left(1\right) & +\mu_{2}(D^{q}y\left(1\right))=\mu_{3}\int_{0}^{1}y\left(\varsigma \right)d\varsigma , \end{array}\right. \end{array}$$ (2.1)

*if and only if y*^⋆^ *is a solution of the integral equations stated below*

$$\begin{array}{ccc} y(s) & = & \int_{0}^{s}\frac{{\left(s-\varsigma \right)}^{p-1}}{\Gamma (p)}\psi \left(\varsigma \right)d\varsigma +(\frac{\mu_{3}}{\mu_{3}-\mu_{1}})\int_{0}^{1}\int_{0}^{\varsigma }\frac{{\left(\varsigma -\tau \right)}^{p-1}}{\Gamma (p)}\psi \left(\tau \right)d\tau d\varsigma \\ & & +(\frac{\Gamma \left(2-q\right)(\mu_{3}-2t\left(\mu_{3}-\mu_{1}\right))}{2\left(\mu_{3}-\mu_{1}\right)\left(\mu_{2}+\mu_{1}\Gamma \left(2-q\right)\right)})\times \\ & & [\frac{\mu_{1}}{\Gamma (p)}\int_{0}^{1}{\left(1-\varsigma \right)}^{p-1}\psi \left(\varsigma \right)d\varsigma +\frac{\mu_{2}}{\Gamma (p-q)}\int_{0}^{1}{\left(1-\varsigma \right)}^{p-q-1}\psi \left(\varsigma \right)d\varsigma ]. \end{array}$$ (2.2)

*Proof*. Let $y^{⋆}:\left[0,1\right]\to R$ is a solution of (2.1). Also, there are constants $z_{0},z_{1}\in R$ such that

$$\begin{array}{c} y^{⋆}\left(s\right)=\int_{0}^{s}\frac{{\left(s-\varsigma \right)}^{p-1}}{\Gamma (p)}\psi \left(\varsigma \right)d\varsigma +z_{0}+z_{1}s. \end{array}$$ (2.3)

Using the boundary conditions for (2.1), we have

$$\begin{array}{ccc} z_{0} & = & (\frac{\mu_{3}}{\mu_{3}-\mu_{1}})\int_{0}^{1}\int_{0}^{\varsigma }\frac{{\left(\varsigma -\tau \right)}^{p-1}}{\Gamma (p)}\psi \left(\tau \right)d\tau d\varsigma -(\frac{\mu_{1}\mu_{3}\Gamma \left(2-q\right)}{2\left(\mu_{3}-\mu_{1}\right)\left(\mu_{2}+\mu_{1}\Gamma \left(2-q\right)\right)})\times \\ & & \{\int_{0}^{1}\frac{{\left(1-\varsigma \right)}^{p-1}}{\Gamma (p)}\psi \left(\varsigma \right)d\varsigma +\int_{0}^{1}\frac{{\left(1-\varsigma \right)}^{p-q-1}}{\Gamma (p-q)}\psi \left(\varsigma \right)d\varsigma \},\\ z_{1} & = & (\frac{\Gamma (2-q)}{\mu_{1}\Gamma \left(2-q\right)+\mu_{2}})\{\mu_{1}\int_{0}^{1}\frac{{\left(1-\varsigma \right)}^{p-1}}{\Gamma (p)}\psi \left(\varsigma \right)d\varsigma +\mu_{2}\int_{0}^{1}\frac{{\left(1-\varsigma \right)}^{p-q-1}}{\Gamma (p-q)}\psi \left(\varsigma \right)d\varsigma \}. \end{array}$$

Substituting the values of *z*_0_ and *z*_1_ in (2.3), we get the solution (2.2). On the converse part, it is clear that *y*^⋆^ can be consider as a solution for (2.1) if *y*^⋆^ is a solution of (2.3).

We now present the Krasnoselskii and Schaefer fixed point theorems, respectively.

**Theorem 2.3** ([56]). *Let* P *be a closed, bounded, convex, and nonempty subset of a Banach space* B *and* $U_{1},U_{2}:P\to B$ *are two operators satisfying the following conditions*:

1.
$U_{1}a+U_{2}b\in P$ for all a,b∈P;
2.
$U_{1}$ *is compact and continuous on* P ;
3.
$U_{2}$ *is a contraction mapping on* P , *that is, there is an* ϱ∈[0,1) *such that*
  $$\begin{array}{c} ∥U_{2}a-U_{2}b∥\leq \varrho ∥a-b∥ \end{array}$$
  *for all* a,b∈P.

*Then* $U_{1}+U_{2}$ *has a fixed point*.

**Theorem 2.4** ([56]). *Let* B *be a Banach space. If* U:B→B *is a completely continuous function, that is*, U *is continuous and totally bounded, then either the set* {a∈B:a=ηUaforsomeη∈(0,1)} *is unbounded or* U *has at least one fixed point in* B.

## 3 Main results

We define the Banach space $\tilde{B}=\left\{y:\left[0,1\right]\to R:y,D^{q}y,y^{\prime },y^{\prime \prime }\in C([0,1],R)\right\}$ having the norm

$$\begin{array}{c} {∥y∥}_{\tilde{B}}=\underset{s\in [0,1]}{\text{sup}}|y(s)|+\underset{s\in [0,1]}{\text{sup}}|D^{q}y\left(s\right)|+\underset{s\in [0,1]}{\text{sup}}|y^{\prime }\left(s\right)|+\underset{s\in [0,1]}{\text{sup}}|y^{\prime \prime }\left(s\right)|. \end{array}$$

Furthermore, it is obvious that $B={\tilde{B}}^{25}$ is a Banach space with

$$\begin{array}{c} {∥y=(y_{1},y_{2},\ldots ,y_{25})∥}_{B}=\sum_{k=1}^{25}{∥y_{k}∥}_{\tilde{B}}. \end{array}$$

Also, by addressing Lemma 2.2, we can define an operator U:B→B for each $(y_{1},y_{2},\ldots ,y_{25})\in B$ by

$$\begin{array}{c} U\left(y_{1},y_{2},\ldots ,y_{25}\right)≔(U_{1}\left(y_{1},y_{2},\ldots ,y_{25}\right),U_{2}\left(y_{1},y_{2},\ldots ,y_{25}\right),\ldots ,U_{25}\left(y_{1},y_{2},\ldots ,y_{25}\right)), \end{array}$$ (3.1)

where for each *k* = 1, 2, …, 25, $U_{k}:B\to \tilde{B}$ is defined for each $(y_{1},y_{2},\ldots ,y_{k})\in B$ by

$$\begin{array}{ccc} & & U_{k}\left(y_{1},y_{2},\ldots ,y_{25}\right)\left(s\right)\\ & = & \int_{0}^{s}\frac{{\left(s-\varsigma \right)}^{p-1}}{\Gamma (p)}Z_{k}\left(\varsigma ,y_{k}\left(\varsigma \right),D^{q}y_{k}\left(\varsigma \right),y_{k}^{\prime }\left(\varsigma \right),y_{k}^{\prime \prime }\left(\varsigma \right)\right)d\varsigma \\ & & +(\frac{\mu_{3}}{\mu_{3}-\mu_{1}})\int_{0}^{1}\int_{0}^{\varsigma }\frac{{\left(\varsigma -\tau \right)}^{p-1}}{\Gamma (p)}Z_{k}(\tau ,y_{k}\left(\tau \right),D^{q}y_{k}\left(\tau \right),y_{k}^{\prime }\left(\tau \right),\\ & & y_{k}^{\prime \prime }\left(\tau \right))d\tau d\varsigma +(\frac{\Gamma \left(2-q\right)(\mu_{3}-2t\left(\mu_{3}-\mu_{1}\right))}{2\left(\mu_{3}-\mu_{1}\right)\left(\mu_{2}+\mu_{1}\Gamma \left(2-q\right)\right)})\times \\ & & [\frac{\mu_{1}}{\Gamma (p)}\int_{0}^{1}{\left(1-\varsigma \right)}^{p-1}Z_{k}\left(\varsigma ,y_{k}\left(\varsigma \right),D^{q}y_{k}\left(\varsigma \right),y_{k}^{\prime }\left(\varsigma \right),y_{k}^{\prime \prime }\left(\varsigma \right)\right))d\varsigma \\ & & +\frac{\mu_{2}}{\Gamma (p-q)}\int_{0}^{1}{\left(1-\varsigma \right)}^{p-q-1}Z_{k}\left(\varsigma ,y_{k}\left(\varsigma \right),D^{q}y_{k}\left(\varsigma \right),y_{k}^{\prime }\left(\varsigma \right),y_{k}^{\prime \prime }\left(\varsigma \right)\right)d\varsigma ] \end{array}$$ (3.2)

for all *s* ∈ [0, 1].

For the ease of calculations, we use the following abbreviations:

$$\begin{array}{ccc} M_{0}^{*} & = & \frac{\left(p+1\right)+\frac{|\mu_{3}|}{|\mu_{3}-\mu_{1}|}}{\Gamma (p+2)}+(\frac{\Gamma \left(2-q\right)|2\mu_{1}-\mu_{3}|}{|2\left(\mu_{3}-\mu_{1}\right)\left(\mu_{2}+\mu_{1}\Gamma \left(2-q\right)\right)|})\times \\ & & (\frac{|\mu_{1}|}{\Gamma (p+1)}+\frac{|\mu_{2}|}{\Gamma (p-q+1)}), \end{array}$$ (3.3)

$$\begin{array}{ccc} M_{1}^{*} & = & \frac{1}{\Gamma (p-q+1)}+\frac{1}{\Gamma \left(2-q\right)|\mu_{2}+\mu_{1}\Gamma \left(2-q\right)|}(\frac{|\mu_{1}|}{\Gamma (p+1)}+\frac{|\mu_{2}|}{\Gamma (p-q+1)}), \end{array}$$ (3.4)

$$\begin{array}{ccc} M_{2}^{*} & = & \frac{1}{\Gamma (p)}+\frac{1}{|\mu_{2}+\mu_{1}\Gamma \left(2-q\right)|}(\frac{|\mu_{1}|}{\Gamma (p+1)}+\frac{|\mu_{2}|}{\Gamma (p-q+1)}), \end{array}$$ (3.5)

$$\begin{array}{ccc} M_{3}^{*} & = & \frac{1}{\Gamma (p-1)}, \end{array}$$ (3.6)

$$\begin{array}{ccc} L_{0}^{*} & = & \frac{|\mu_{3}|}{\Gamma \left(p+2\right)|\mu_{3}-\mu_{1}|}+(\frac{\Gamma \left(2-q\right)|2\mu_{1}-\mu_{3}|}{|2\left(\mu_{3}-\mu_{1}\right)\left(\mu_{2}+\mu_{1}\Gamma \left(2-q\right)\right)|})\times \\ & & (\frac{|\mu_{1}|}{\Gamma (p+1)}+\frac{|\mu_{2}|}{\Gamma (p-q+1)}), \end{array}$$ (3.7)

$$\begin{array}{ccc} L_{1}^{*} & = & \frac{1}{\Gamma \left(2-q\right)|\mu_{2}+\mu_{1}\Gamma \left(2-q\right)|}(\frac{|\mu_{1}|}{\Gamma (p+1)}+\frac{|\mu_{2}|}{\Gamma (p-q+1)}), \end{array}$$ (3.8)

$$\begin{array}{ccc} L_{2}^{*} & = & \frac{1}{|\mu_{2}+\mu_{1}\Gamma \left(2-q\right)|}(\frac{|\mu_{1}|}{\Gamma (p+1)}+\frac{|\mu_{2}|}{\Gamma (p-q+1)}). \end{array}$$ (3.9)

**Theorem 3.1** *Consider the fractional boundary value problem* (1.1). *Assume that* $Z_{1},Z_{2},\ldots ,Z_{25}:\left[0,1\right]\times R\times R\times R\times R\to R$ *are continuous functions and there are constants* $Q_{k}>0$, *for all k* = 1, 2, …, 25 *with*

$$\begin{array}{c} |Z_{k}\left(s,z_{1},z_{2},z_{3},z_{4}\right)|\leq Q_{k} \end{array}$$

*for all* $z_{1},z_{2},z_{3},z_{4}\in R$, *s* ∈ [0, 1]. *Then* (1.1) *has a solution*.

*Proof*. The fixed points of U given in (3.1) exist if and only if (1.1) has a solution, as shown by the consequence of (3.2). To demonstrate this, we must first prove that U is completely continuous.

As $Z_{1},Z_{2},\ldots ,Z_{25}$ are continuous, therefore U:B→B is continuous too. Let O∈B be a bounded set and $y=(y_{1},y_{2},...,y_{25})\in B$, so for each *s* ∈ [0, 1], we have

$$\begin{array}{ccc} & & \left|\left(U_{k}y\right)\left(s\right)\right|\\ & \leq & \int_{0}^{s}\frac{{\left(s-\varsigma \right)}^{p-1}}{\Gamma (p)}\left|Z_{k}\left(\varsigma ,y_{k}\left(\varsigma \right),D^{q}y_{k}\left(\varsigma \right),y_{k}^{\prime }\left(\varsigma \right),y_{k}^{\prime \prime }\left(\varsigma \right)\right)\right|d\varsigma +\frac{|\mu_{3}|}{|\mu_{3}-\mu_{1}|}\int_{0}^{1}\int_{0}^{\varsigma }\frac{{\left(\varsigma -\tau \right)}^{p-1}}{\Gamma (p)}\\ & & \times |Z_{k}\left(\tau ,y_{k}\left(\tau \right),D^{q}y_{k}\left(\tau \right),y_{k}^{\prime }\left(\tau \right),y_{k}^{\prime \prime }\left(\tau \right)\right)|d\tau d\varsigma +\frac{\Gamma \left(2-q\right)|\mu_{3}-2t\left(\mu_{3}-\mu_{1}\right)|}{|2\left(\mu_{3}-\mu_{1}\right)\left(\mu_{2}+\mu_{1}\Gamma \left(2-q\right)\right)|}\times \\ & & [\frac{|\mu_{1}|}{\Gamma (p)}\int_{0}^{1}{\left(1-\varsigma \right)}^{p-1}|Z_{k}\left(\varsigma ,y_{k}\left(\varsigma \right),D^{q}y_{k}\left(\varsigma \right),y_{k}^{\prime }\left(\varsigma \right),y_{k}^{\prime \prime }\left(\varsigma \right)\right))|d\varsigma +\frac{|\mu_{2}|}{\Gamma (p-q)}\\ & & \times \int_{0}^{1}{\left(1-\varsigma \right)}^{p-q-1}\left|Z_{k}\left(\varsigma ,y_{k}\left(\varsigma \right),D^{q}y_{k}\left(\varsigma \right),y_{k}^{\prime }\left(\varsigma \right),y_{k}^{\prime \prime }\left(\varsigma \right)\right)\right|d\varsigma ]\\ & \leq & \int_{0}^{s}\frac{{\left(s-\varsigma \right)}^{p-1}}{\Gamma (p)}Q_{k}d\varsigma +\frac{|\mu_{3}|}{|\mu_{3}-\mu_{1}|}\int_{0}^{1}\int_{0}^{\varsigma }\frac{{\left(\varsigma -\tau \right)}^{p-1}}{\Gamma (p)}Q_{k}d\tau d\varsigma +\frac{\Gamma (2-q)}{2|\mu_{3}-\mu_{1}|}\\ & & +\frac{|\mu_{3}-2t\left(\mu_{3}-\mu_{1}\right)|}{|\mu_{2}+\mu_{1}\Gamma \left(2-q\right)|}\times [\frac{|\mu_{1}|}{\Gamma (p)}\int_{0}^{1}{\left(1-\varsigma \right)}^{p-1}Q_{k}d\varsigma +\frac{|\mu_{2}|}{\Gamma (p-q)}\int_{0}^{1}{\left(1-\varsigma \right)}^{p-q-1}Q_{k}d\varsigma ]\\ & \leq & Q_{k}[\frac{\left(p+1\right)+\frac{|\mu_{3}|}{|\mu_{3}-\mu_{1}|}}{\Gamma (p+2)}+\frac{\Gamma \left(2-q\right)|2\mu_{1}-\mu_{3}|}{|2\left(\mu_{3}-\mu_{1}\right)\left(\mu_{2}+\mu_{1}\Gamma \left(2-q\right)\right)|}(\frac{|\mu_{1}|}{\Gamma (p+1)}+\frac{|\mu_{2}|}{\Gamma (p-q+1)})]\\ & = & Q_{k}M_{0}^{*}, \end{array}$$

where $M_{0}^{*}$ is given in (3.3). Also,

$$\begin{array}{ccc} & & \left|\left(D^{q}U_{k}y\right)\left(s\right)\right|\\ & \leq & \int_{0}^{s}\frac{{\left(s-\varsigma \right)}^{p-q}}{\Gamma (p-q)}\left|Z_{k}\left(\varsigma ,y_{k}\left(\varsigma \right),D^{q}y_{k}\left(\varsigma \right),y_{k}^{\prime }\left(\varsigma \right),y_{k}^{\prime \prime }\left(\varsigma \right)\right)\right|d\varsigma +\frac{s^{1-q}}{\Gamma \left(2-q\right)|\mu_{2}+\mu_{1}\Gamma \left(2-q\right)|}\\ & & \times [\frac{|\mu_{1}|}{\Gamma (p)}\int_{0}^{1}{\left(1-\varsigma \right)}^{p-1}|Z_{k}\left(\varsigma ,y_{k}\left(\varsigma \right),D^{q}y_{k}\left(\varsigma \right),y_{k}^{\prime }\left(\varsigma \right),y_{k}^{\prime \prime }\left(\varsigma \right)\right))|d\varsigma +\frac{|\mu_{2}|}{\Gamma (p-q)}\times \\ & & \int_{0}^{1}{\left(1-\varsigma \right)}^{p-q-1}\left|Z_{k}\left(\varsigma ,y_{k}\left(\varsigma \right),D^{q}y_{k}\left(\varsigma \right),y_{k}^{\prime }\left(\varsigma \right),y_{k}^{\prime \prime }\left(\varsigma \right)\right)\right|d\varsigma ]\\ & \leq & Q_{k}[\frac{1}{\Gamma (p-q+1)}+\frac{1}{\Gamma \left(2-q\right)|\mu_{2}+\mu_{1}\Gamma \left(2-q\right)|}(\frac{|\mu_{1}|}{\Gamma (p+1)}+\frac{|\mu_{2}|}{\Gamma (p-q+1)})]\\ & = & Q_{k}M_{1}^{*} \end{array}$$

and

$$\begin{array}{ccc} \left|\left(U_{k}^{\prime }y\right)\left(s\right)\right| & \leq & \int_{0}^{s}\frac{{\left(s-\varsigma \right)}^{p-2}}{\Gamma (p-1)}\left|Z_{k}\left(\varsigma ,y_{k}\left(\varsigma \right),D^{q}y_{k}\left(\varsigma \right),y_{k}^{\prime }\left(\varsigma \right),y_{k}^{\prime \prime }\left(\varsigma \right)\right)\right|d\varsigma +\frac{1}{|\mu_{2}+\mu_{1}\Gamma \left(2-q\right)|}\\ & & \times [\frac{|\mu_{1}|}{\Gamma (p)}\int_{0}^{1}{\left(1-\varsigma \right)}^{p-1}|Z_{k}\left(\varsigma ,y_{k}\left(\varsigma \right),D^{q}y_{k}\left(\varsigma \right),y_{k}^{\prime }\left(\varsigma \right),y_{k}^{\prime \prime }\left(\varsigma \right)\right))|d\varsigma \\ & & +\frac{|\mu_{2}|}{\Gamma (p-q)}\int_{0}^{1}{\left(1-\varsigma \right)}^{p-q-1}\left|Z_{k}\left(\varsigma ,y_{k}\left(\varsigma \right),D^{q}y_{k}\left(\varsigma \right),y_{k}^{\prime }\left(\varsigma \right),y_{k}^{\prime \prime }\left(\varsigma \right)\right)\right|d\varsigma ]\\ & \leq & Q_{k}[\frac{1}{\Gamma (p)}+\frac{1}{|\mu_{2}+\mu_{1}\Gamma \left(2-q\right)|}(\frac{|\mu_{1}|}{\Gamma (p+1)}+\frac{|\mu_{2}|}{\Gamma (p-q+1)})]\\ & = & Q_{k}M_{2}^{*} \end{array}$$

for all *s* ∈ [0, 1], where $M_{1}^{*}$ and $M_{2}^{*}$ are defined in (3.4) and (3.5), respectively. Similarly,

$$\begin{array}{ccc} \left|\left(U_{k}^{\prime \prime }y\right)\left(s\right)\right| & \leq & Q_{k}M_{3}^{*} \end{array}$$

for all *s* ∈ [0, 1], where $M_{3}^{*}$ is given in (3.6). Therefore

$$\begin{array}{c} {∥U_{k}y∥}_{\tilde{B}}\leq Q_{k}(M_{0}^{*}+M_{1}^{*}+M_{2}^{*}+M_{3}^{*}). \end{array}$$

Hence,

$$\begin{array}{ccc} {∥Uy∥}_{B} & = & \sum_{k=1}^{25}{∥U_{k}y∥}_{\tilde{B}}\\ & \leq & \sum_{k=1}^{25}Q_{k}(M_{0}^{*}+M_{1}^{*}+M_{2}^{*}+M_{3}^{*})\\ & < & \infty , \end{array}$$

which reveals that U is uniformly bounded.

To prove the equicontinuity of the operator U, we let $y=(y_{1},y_{2},\ldots ,y_{25})\in O$ and *s*_1_, *s*_2_ ∈ [0, 1] with *s*_1_ < *s*_2_. Then we have

$$\begin{array}{ccc} & & \left|\left(U_{k}y\right)\left(s_{2}\right)-\left(U_{k}y\right)\left(s_{1}\right)\right|\\ & = & \int_{0}^{s_{1}}\frac{{\left(s_{2}-\varsigma \right)}^{p-1}-{\left(s_{1}-\varsigma \right)}^{p-1}}{\Gamma (p)}\left|Z_{k}\left(\varsigma ,y_{k}\left(\varsigma \right),D^{q}y_{k}\left(\varsigma \right),y_{k}^{\prime }\left(\varsigma \right),y_{k}^{\prime \prime }\left(\varsigma \right)\right)\right|d\varsigma \\ & & +\int_{s_{1}}^{s_{2}}\frac{{\left(s_{2}-\varsigma \right)}^{p-1}}{\Gamma (p)}\left|Z_{k}\left(\varsigma ,y_{k}\left(\varsigma \right),D^{q}y_{k}\left(\varsigma \right),y_{k}^{\prime }\left(\varsigma \right),y_{k}^{\prime \prime }\left(\varsigma \right)\right)\right|d\varsigma \frac{s_{2}-s_{1}}{|\mu_{2}+\mu_{1}\Gamma \left(2-q\right)|}\times \\ & & +[\frac{|\mu_{1}|}{\Gamma (p)}\int_{0}^{1}{\left(1-\varsigma \right)}^{p-1}|Z_{k}\left(\varsigma ,y_{k}\left(\varsigma \right),D^{q}y_{k}\left(\varsigma \right),y_{k}^{\prime }\left(\varsigma \right),y_{k}^{\prime \prime }\left(\varsigma \right)\right))|d\varsigma +\frac{|\mu_{2}|}{\Gamma (p-q)}\times \\ & & \int_{0}^{1}{\left(1-\varsigma \right)}^{p-q-1}\left|Z_{k}\left(\varsigma ,y_{k}\left(\varsigma \right),D^{q}y_{k}\left(\varsigma \right),y_{k}^{\prime }\left(\varsigma \right),y_{k}^{\prime \prime }\left(\varsigma \right)\right)\right|d\varsigma ]. \end{array}$$

It is clear that if *s*_1_ → *s*_2_ then, independently, the right-hand side of the above equation converges to zero. Also

$$\begin{array}{c} \underset{s_{1}\to s_{2}}{\text{lim}}|\left(D^{q}U_{k}y\right)\left(s_{2}\right)-\left(D^{q}U_{k}y\right)\left(s_{1}\right)|=0, \end{array}$$

and

$$\begin{array}{c} \underset{s_{1}\to s_{2}}{\text{lim}}|\left(U_{k}^{\prime }y\right)\left(s_{2}\right)-\left(U_{k}^{\prime }y\right)\left(s_{1}\right)|=0,\;\underset{s_{1}\to s_{2}}{\text{lim}}|\left(U_{k}^{\prime \prime }y\right)\left(s_{2}\right)-\left(U_{k}^{\prime \prime }y\right)\left(s_{1}\right)|=0. \end{array}$$

Hence, we deduce that the operators $U_{k}\;\left(k=1,2,\ldots ,25\right)$ are equicontinuous, which implies that U is equicontinuous. The Arzela–Ascoli theorem now entails the complete continuity of the operator.

Further, we define a set

$$\begin{array}{c} ϒ≔\{\left(y_{1},y_{2},\ldots ,y_{25}\right)\in B:\left(y_{1},y_{2},\ldots ,y_{25}\right)=\eta U\left(y_{1},y_{2},\ldots ,y_{25}\right),\;\eta \in \left(0,1\right)\} \end{array}$$

on B. Now, we will prove that Υ is bounded. For this, let (*y*_1_, *y*_2_, …, *y*_25_)∈Υ. Then, we can write

$$\begin{array}{c} \left(y_{1},y_{2},\ldots ,y_{25}\right)=\eta U\left(y_{1},y_{2},\ldots ,y_{25}\right), \end{array}$$

and so

$$\begin{array}{c} y_{k}\left(s\right)=\eta \left(U\left(y_{1},y_{2},\ldots ,y_{25}\right)\right)\left(s\right), \end{array}$$

for all *s* ∈ [0, 1] and *k* = 1, 2, …, 25. Thus,

$$\begin{array}{ccc} & & \left|y_{k}\left(s\right)\right|\\ & \leq & \eta [\int_{0}^{s}\frac{{\left(s-\varsigma \right)}^{p-1}}{\Gamma (p)}\left|Z_{k}\left(\varsigma ,y_{k}\left(\varsigma \right),D^{q}y_{k}\left(\varsigma \right),y_{k}^{\prime }\left(\varsigma \right),y_{k}^{\prime \prime }\left(\varsigma \right)\right)\right|d\varsigma +\frac{|\mu_{3}|}{|\mu_{3}-\mu_{1}|}\int_{0}^{1}\int_{0}^{\varsigma }\frac{{\left(\varsigma -\tau \right)}^{p-1}}{\Gamma (p)}\\ & & |Z_{k}\left(\tau ,y_{k}\left(\tau \right),D^{q}y_{k}\left(\tau \right),y_{k}^{\prime }\left(\tau \right),y_{k}^{\prime \prime }\left(\tau \right)\right)|d\tau d\varsigma +\frac{\Gamma \left(2-q\right)|\mu_{3}-2t\left(\mu_{3}-\mu_{1}\right)|}{|2\left(\mu_{3}-\mu_{1}\right)\left(\mu_{2}+\mu_{1}\Gamma \left(2-m\right)\right)|}\times \\ & & \{\frac{|\mu_{1}|}{\Gamma (p)}\int_{0}^{1}{\left(1-\varsigma \right)}^{p-1}|Z_{k}\left(\varsigma ,y_{k}\left(\varsigma \right),D^{q}y_{k}\left(\varsigma \right),y_{k}^{\prime }\left(\varsigma \right),y_{k}^{\prime \prime }\left(\varsigma \right)\right))|d\varsigma \\ & & +\frac{|\mu_{2}|}{\Gamma (p-q)}\int_{0}^{1}{\left(1-\varsigma \right)}^{p-q-1}\left|Z_{k}\left(\varsigma ,y_{k}\left(\varsigma \right),D^{q}y_{k}\left(\varsigma \right),y_{k}^{\prime }\left(\varsigma \right),y_{k}^{\prime \prime }\left(\varsigma \right)\right)\right|d\varsigma \}]\\ & \leq & \eta Q_{k}M_{0}^{*}, \end{array}$$

and by similar computations, we have

$$\begin{array}{ccc} \left|D^{q}y_{k}\left(s\right)\right| & \leq & \eta Q_{k}M_{1}^{*},\\ \left|y_{k}^{\prime }\left(s\right)\right| & \leq & \eta Q_{k}M_{2}^{*},\\ \left|y_{k}^{\prime \prime }\left(s\right)\right| & \leq & \eta Q_{k}M_{3}^{*}, \end{array}$$

where $M_{0}^{*}-M_{3}^{*}$ are given in (3.3)–(3.6). Hence,

$$\begin{array}{ccc} {∥y=(y_{1},y_{2},\ldots ,y_{25})∥}_{B} & = & \sum_{k=1}^{25}{∥y_{k}∥}_{\tilde{B}}\\ & \leq & \eta \sum_{k=1}^{25}Q_{k}(M_{0}^{*}+M_{1}^{*}+M_{2}^{*}+M_{3}^{*})\\ & < & \infty , \end{array}$$

which demonstrates the boundedness of the operator Υ. Now, using Lemma 2.2 and Theorem 2.4, it is clear that the operator U has a fixed point. Consequently, (1.1) does indeed have a solution.

We shall now investigate the solution of (1.1) by applying various conditions.

**Theorem 3.2** *Consider the fractional boundary value problem* (1.1). *Suppose that* $Z_{1},\ldots ,Z_{25}:\left[0,1\right]\times R\times R\times R\times R\to R$ *are continuous functions and there are bounded continuous functions* $K_{1},\ldots ,K_{25}:\left[0,1\right]\to R$, $R_{1},\ldots ,R_{25}:\left[0,1\right]\to \left[0,\infty \right)$ *and nondecreasing continuous functions σ*_1_, …, *σ*_25_: [0, 1] → [0, ∞] *such that*

$$\begin{array}{c} |Z_{k}\left(s,z_{1},z_{2},z_{3},z_{4}\right)|\leq R_{k}\left(s\right)\sigma_{k}\left(|z_{1}|+|z_{2}|+|z_{3}|+|z_{4}|\right) \end{array}$$

*and*

$$\begin{array}{c} |Z_{k}\left(s,z_{1}^{⋆},z_{2}^{⋆},z_{3}^{⋆},z_{4}^{⋆}\right)-Z_{k}\left(s,z_{1},z_{2},z_{3},z_{4}\right)|\leq K_{k}\left(s\right)\left(|z_{1}^{⋆}-z_{1}|+|z_{2}^{⋆}-z_{2}|+|z_{3}^{⋆}-z_{3}|+|z_{4}^{⋆}-z_{4}|\right)\; \end{array}$$

*for all s* ∈ [0, 1], $z_{1}^{⋆},z_{2}^{⋆},z_{3}^{⋆},z_{4}^{⋆},z_{1},z_{2},z_{3},z_{4}\in R$ *and k* = 1, 2, …, 25. *If*

$$\begin{array}{c} M≔(L_{0}^{*}+L_{1}^{*}+L_{2}^{*})\sum_{k=1}^{25}∥K_{k}∥<1, \end{array}$$

*then* (1.1) *has a solution, where* $∥K_{k}∥={\text{sup}}_{s\in [0,1]}|K_{k}\left(s\right)|$ *and the constants* $L_{0}^{*}$–$L_{2}^{*}$ *are given in* (3.7)–(3.9), *respectively*.

*Proof*. For each *k* = 1, 2, …25, let $∥R_{k}∥={\text{sup}}_{s\in [0,1]}|R_{k}\left(s\right)|$ and for suitable constants $\varpi_{k}$, we have

$$\begin{array}{c} \varpi_{k}\geq \sum_{k=1}^{25}\sigma_{i}({∥y_{k}∥}_{B_{k}})∥R_{k}∥\{M_{0}^{*}+M_{1}^{*}+M_{2}^{*}+M_{3}^{*}\}, \end{array}$$ (3.10)

where $M_{0}^{*}-M_{3}^{*}$ are given in (3.3)–(3.6). We define a set

$$\begin{array}{c} O_{\varpi_{k}}≔\{y=\left(y_{1},y_{2},\ldots ,y_{25}\right)\in B:{∥y∥}_{B}\leq \varpi_{k}\}, \end{array}$$

where $\varpi_{k}$ is defined in (3.10). It is obvious that $O_{\varpi_{k}}$ be a closed, nonempty, bounded, and convex subset of $B=B_{1}\times B_{2}\times \ldots \times B_{25}$. Now, we have $U_{1}$ and $U_{2}$ which are define on $O_{\varpi_{k}}$ as

$$\begin{array}{ccc} U_{1}\left(y_{1},y_{2},\ldots ,y_{25}\right)\left(s\right) & ≔ & (U_{1}^{\left(1\right)}\left(y_{1},y_{2},\ldots ,y_{25}\right)\left(s\right),\ldots ,U_{1}^{\left(25\right)}\left(y_{1},y_{2},\ldots ,y_{25}\right)\left(s\right)),\\ U_{2}\left(y_{1},y_{2},\ldots ,y_{25}\right)\left(s\right) & ≔ & (U_{2}^{\left(1\right)}\left(y_{1},y_{2},\ldots ,y_{25}\right)\left(s\right),\ldots ,U_{2}^{\left(25\right)}\left(y_{1},y_{2},\ldots ,y_{25}\right)\left(s\right)), \end{array}$$

where

$$\begin{array}{c} (U_{1}^{\left(k\right)}y)\left(s\right)=\int_{0}^{s}\frac{{\left(s-\varsigma \right)}^{p-1}}{\Gamma (p)}Z_{k}\left(\varsigma ,y_{k}\left(\varsigma \right),D^{q}y_{k}\left(\varsigma \right),y_{k}^{\prime }\left(\varsigma \right),y_{k}^{\prime \prime }\left(\varsigma \right)\right)d\varsigma \end{array}$$ (3.11)

and

$$\begin{array}{ccc} & & (U_{2}^{\left(k\right)}y)\left(s\right)\\ & = & (\frac{\mu_{3}}{\mu_{3}-\mu_{1}})\int_{0}^{1}\int_{0}^{\varsigma }\frac{{\left(\varsigma -\tau \right)}^{p-1}}{\Gamma (p)}Z_{k}\left(\tau ,y_{k}\left(\tau \right),D^{q}y_{k}\left(\tau \right),y_{k}^{\prime }\left(\tau \right),y_{k}^{\prime \prime }\left(\tau \right)\right)d\tau d\varsigma \\ & & +(\frac{\Gamma \left(2-q\right)\mu_{3}-2t\left(\mu_{3}-\mu_{1}\right)}{2\left(\mu_{3}-\mu_{1}\right)\left(\mu_{2}+\mu_{1}\Gamma \left(2-q\right)\right)})\times \\ & & [\frac{\mu_{1}}{\Gamma (p)}\int_{0}^{1}{\left(1-\varsigma \right)}^{p-1}Z_{k}\left(\varsigma ,y_{k}\left(\varsigma \right),D^{q}y_{k}\left(\varsigma \right),y_{k}^{\prime }\left(\varsigma \right),y_{k}^{\prime \prime }\left(\varsigma \right)\right))d\varsigma \\ & & +\frac{\mu_{2}}{\Gamma (p-q)}\int_{0}^{1}{\left(1-\varsigma \right)}^{p-q-1}Z_{k}\left(\varsigma ,y_{k}\left(\varsigma \right),D^{q}y_{k}\left(\varsigma \right),y_{k}^{\prime }\left(\varsigma \right),y_{k}^{\prime \prime }\left(\varsigma \right)\right)d\varsigma ] \end{array}$$ (3.12)

for all *s* ∈ [0, 1] and *y* = $\left(y_{1},y_{2},\ldots ,y_{25}\right)\in O_{\varpi_{k}}.$

Let ${\tilde{\sigma }}_{k}={\text{sup}}_{y_{k}\in B_{k}}\sigma_{k}({∥y_{k}∥}_{B_{k}}).$ Now, for every $z=\left(z_{1},z_{2},\ldots ,z_{25}\right),y=\left(y_{1},y_{2},\ldots ,y_{25}\right)\in O_{\varpi_{k}},$ we have

$$\begin{array}{ccc} & & \left|(U_{1}^{\left(k\right)}z+U_{2}^{\left(k\right)}y)\left(s\right)\right|\\ & \leq & \int_{0}^{s}\frac{{\left(s-\varsigma \right)}^{p-1}}{\Gamma (p)}\left|Z_{k}\left(\varsigma ,z_{k}\left(\varsigma \right),D^{q}z_{k}\left(\varsigma \right),z_{k}^{\prime }\left(\varsigma \right),z_{k}^{\prime \prime }\left(\varsigma \right)\right)\right|d\varsigma +\frac{|\mu_{3}|}{|\mu_{3}-\mu_{1}|}\int_{0}^{1}\int_{0}^{\varsigma }\frac{{\left(\varsigma -\tau \right)}^{p-1}}{\Gamma (p)}\\ & & |Z_{k}\left(\tau ,y_{k}\left(\tau \right),D^{q}y_{k}\left(\tau \right),y_{k}^{\prime }\left(\tau \right),y_{k}^{\prime \prime }\left(\tau \right)\right)|d\tau d\varsigma +\frac{\Gamma \left(2-q\right)|\mu_{3}-2t\left(\mu_{3}-\mu_{1}\right)|}{|2\left(\mu_{3}-\mu_{1}\right)\left(\mu_{2}+\mu_{1}\Gamma \left(2-q\right)\right)|}\times \\ & & [\frac{|\mu_{1}|}{\Gamma (p)}\int_{0}^{1}{\left(1-\varsigma \right)}^{p-1}|Z_{k}\left(\varsigma ,y_{k}\left(\varsigma \right),D^{q}y_{k}\left(\varsigma \right),y_{k}^{\prime }\left(\varsigma \right),y_{k}^{\prime \prime }\left(\varsigma \right)\right))|d\varsigma +\frac{|\mu_{2}|}{\Gamma (p-q)}\\ & & \int_{0}^{1}{\left(1-\varsigma \right)}^{p-q-1}\left|Z_{k}\left(\varsigma ,y_{k}\left(\varsigma \right),D^{q}y_{k}\left(\varsigma \right),y_{k}^{\prime }\left(\varsigma \right),y_{k}^{\prime \prime }\left(\varsigma \right)\right)\right|d\varsigma ]\\ & \leq & \int_{0}^{s}\frac{{\left(s-\varsigma \right)}^{p-1}}{\Gamma (p)}R_{k}\left(\varsigma \right)\sigma_{k}(|z_{k}\left(\varsigma \right)|+|D^{q}z_{k}\left(\varsigma \right)|+|z_{k}^{\prime }\left(\varsigma \right)|+|z_{k}^{\prime \prime }\left(\varsigma \right)|)d\varsigma +\frac{|\mu_{3}|}{|\mu_{3}-\mu_{1}|}\\ & & \int_{0}^{1}\int_{0}^{\varsigma }\frac{{\left(\varsigma -\tau \right)}^{p-1}}{\Gamma (p)}R_{k}\left(\tau \right)\sigma_{k}(|y_{k}\left(\tau \right)|+|D^{q}y_{k}\left(\tau \right)|+|y_{k}^{\prime }\left(\tau \right)|+|y_{k}^{\prime \prime }\left(\tau \right)|)d\tau d\varsigma \\ & & +\frac{\Gamma \left(2-q\right)|\mu_{3}-2t\left(\mu_{3}-\mu_{1}\right)|}{|2\left(\mu_{3}-\mu_{1}\right)\left(\mu_{2}+\mu_{1}\Gamma \left(2-q\right)\right)|}\times \\ & & [\frac{|\mu_{1}|}{\Gamma (p)}\int_{0}^{1}{\left(1-\varsigma \right)}^{p-1}R_{k}\left(\varsigma \right)\sigma_{k}(|y_{k}\left(\varsigma \right)|+|D^{q}y_{k}\left(\varsigma \right)|+|y_{k}^{\prime }\left(\varsigma \right)|+|y_{k}^{\prime \prime }\left(\varsigma \right)|)d\varsigma \\ & & +\frac{|\mu_{2}|}{\Gamma (p-q)}\int_{0}^{1}{\left(1-\varsigma \right)}^{p-q-1}R_{k}\left(\varsigma \right)\sigma_{k}(|y_{k}\left(\varsigma \right)|+|D^{q}y_{k}\left(\varsigma \right)|+|y_{k}^{\prime }\left(\varsigma \right)|+|y_{k}^{\prime \prime }\left(\varsigma \right)|)d\varsigma ]\\ & \leq & ∥R_{k}∥{\tilde{\sigma }}_{k}[\frac{\left(p+1\right)+\frac{|\mu_{3}|}{|\mu_{3}-\mu_{1}|}}{\Gamma (p+2)}+\frac{\Gamma \left(2-q\right)|2\mu_{1}-\mu_{3}|}{|2\left(\mu_{3}-\mu_{1}\right)\left(\mu_{2}+\mu_{1}\Gamma \left(2-m\right)\right)|}\\ & & \times (\frac{|\mu_{1}|}{\Gamma (p+1)}+\frac{|\mu_{2}|}{\Gamma (p-q+1)})]\\ & = & ∥R_{k}∥{\tilde{\sigma }}_{k}M_{0}^{*}, \end{array}$$

and

$$\begin{array}{ccc} & & \left|D^{q}U_{1}^{\left(k\right)}z\left(s\right)+D^{q}U_{2}^{\left(k\right)}y\left(s\right)\right|\\ & \leq & \int_{0}^{s}\frac{{\left(s-\varsigma \right)}^{p-q-1}}{\Gamma (p-m)}\left|Z_{k}\left(\varsigma ,z_{k}\left(\varsigma \right),D^{q}z_{k}\left(\varsigma \right),z_{k}^{\prime }\left(\varsigma \right),z_{k}^{\prime \prime }\left(\varsigma \right)\right)\right|d\varsigma +\frac{s^{1-q}}{\Gamma \left(2-q\right)|\mu_{2}+\mu_{1}\Gamma \left(2-m\right)|}\\ & & [\frac{|\mu_{1}|}{\Gamma (p)}\int_{0}^{1}{\left(1-\varsigma \right)}^{p-1}|Z_{k}\left(\varsigma ,y_{k}\left(\varsigma \right),D^{q}y_{k}\left(\varsigma \right),y_{k}^{\prime }\left(\varsigma \right),y_{k}^{\prime \prime }\left(\varsigma \right)\right))|d\varsigma \\ & & +\frac{|\mu_{2}|}{\Gamma (p-q)}\int_{0}^{1}{\left(1-\varsigma \right)}^{p-q-1}\left|Z_{k}\left(\varsigma ,y_{k}\left(\varsigma \right),D^{q}y_{k}\left(\varsigma \right),y_{k}^{\prime }\left(\varsigma \right),y_{k}^{\prime \prime }\left(\varsigma \right)\right)\right|d\varsigma ]\\ & \leq & ∥R_{k}∥{\tilde{\sigma }}_{k}[\frac{1}{\Gamma (p-q+1)}+\frac{1}{\Gamma \left(2-q\right)|\mu_{2}+\mu_{1}\Gamma \left(2-q\right)|}(\frac{|\mu_{1}|}{\Gamma (p+1)}+\frac{|\mu_{2}|}{\Gamma (p-q+1)})]\\ & = & ∥R_{k}∥{\tilde{\sigma }}_{k}M_{1}^{*}. \end{array}$$

By using similar computations, we have

$$\begin{array}{ccc} & & \left|{\left(U_{1}^{\left(k\right)}z\right)}^{\prime }\left(s\right)+{\left(U_{2}^{\left(k\right)}y\right)}^{\prime }\left(s\right)\right|\\ & \leq & \int_{0}^{s}\frac{{\left(s-\varsigma \right)}^{p-2}}{\Gamma (p-1)}\left|Z_{k}\left(\varsigma ,z_{k}\left(\varsigma \right),D^{q}z_{k}\left(\varsigma \right),z_{k}^{\prime }\left(\varsigma \right),z_{k}^{\prime \prime }\left(\varsigma \right)\right)\right|d\varsigma +\frac{1}{|\mu_{2}+\mu_{1}\Gamma \left(2-q\right)|}\\ & & [\frac{|\mu_{1}|}{\Gamma (p)}\int_{0}^{1}{\left(1-\varsigma \right)}^{p-1}|Z_{k}\left(\varsigma ,y_{k}\left(\varsigma \right),D^{q}y_{k}\left(\varsigma \right),y_{k}^{\prime }\left(\varsigma \right),y_{k}^{\prime \prime }\left(\varsigma \right)\right))|d\varsigma \\ & & +\frac{|\mu_{2}|}{\Gamma (p-q)}\int_{0}^{1}{\left(1-\varsigma \right)}^{p-q-1}\left|Z_{k}\left(\varsigma ,y_{k}\left(\varsigma \right),D^{q}y_{k}\left(\varsigma \right),y_{k}^{\prime }\left(\varsigma \right),y_{k}^{\prime \prime }\left(\varsigma \right)\right)\right|d\varsigma ]\\ & \leq & ∥R_{k}∥{\tilde{\sigma }}_{k}[\frac{1}{\Gamma (p)}+\frac{1}{|\mu_{2}+\mu_{1}\Gamma \left(2-m\right)|}(\frac{|\mu_{1}|}{\Gamma (p+1)}+\frac{|\mu_{2}|}{\Gamma (p-q+1)})]\\ & = & ∥R_{k}∥{\tilde{\sigma }}_{k}M_{2}^{*}, \end{array}$$

also

$$\begin{array}{ccc} \left|{\left(U_{1}^{\left(k\right)}z\right)}^{\prime \prime }\left(s\right)+{\left(U_{2}^{\left(k\right)}y\right)}^{\prime \prime }\left(s\right)\right| & \leq & ∥R_{k}∥{\tilde{\sigma }}_{k}M_{3}^{*}. \end{array}$$

This yields that

$$\begin{array}{ccc} {∥U_{1}z+U_{2}y∥}_{B} & = & \sum_{k=1}^{25}{∥U_{1}^{\left(k\right)}z+U_{2}^{\left(k\right)}y∥}_{B_{k}}\\ & \leq & ∥R_{k}∥{\tilde{\sigma }}_{k}(M_{0}^{*}+M_{1}^{*}+M_{2}^{*}+M_{3}^{*})\\ & \leq & \varpi_{k}, \end{array}$$

and so $U_{1}z+U_{2}y\in O_{\varpi_{k}}.$ Additionally, continuity of $Z_{k}$ entails the continuity of $U_{1}$.

We now need to demonstrate that $U_{1}$ is uniformly bounded. This is why, we have

$$\begin{array}{ccc} \left|(U_{1}^{\left(k\right)}y)\left(s\right)\right| & \leq & \int_{0}^{s}\frac{{\left(s-\varsigma \right)}^{p-1}}{\Gamma (p)}|Z_{k}\left(\varsigma ,y_{k}\left(\varsigma \right),D^{q}y_{k}\left(\varsigma \right),y_{k}^{\prime }\left(\varsigma \right),y_{k}^{\prime \prime }\left(\varsigma \right)\right))|d\varsigma \\ & \leq & \frac{1}{\Gamma (p+1)}∥R_{k}∥\sigma_{k}(|y_{k}\left(\varsigma \right)|+|D^{q}y_{k}\left(\varsigma \right)|+|y_{k}^{\prime }\left(\varsigma \right)|+|y_{k}^{\prime \prime }\left(\varsigma \right)|). \end{array}$$

for all *y* ∈ $O_{\varpi_{k}}.$ Also,

$$\begin{array}{ccc} \left|(D^{q}U_{1}^{\left(k\right)}y)\left(s\right)\right| & \leq & \int_{0}^{s}\frac{{\left(s-\varsigma \right)}^{p-q-1}}{\Gamma (p-m)}\left|Z_{k}\left(\varsigma ,y_{k}\left(\varsigma \right),D^{q}y_{k}\left(\varsigma \right),y_{k}^{\prime }\left(\varsigma \right),y_{k}^{\prime \prime }\left(\varsigma \right)\right)\right|d\varsigma \\ & \leq & \frac{1}{\Gamma (p-q+1)}∥R_{k}∥\sigma_{k}(|y_{k}\left(\varsigma \right)|+|D^{q}y_{k}\left(\varsigma \right)|+|y_{k}^{\prime }\left(\varsigma \right)|+|y_{k}^{\prime \prime }\left(\varsigma \right)|), \end{array}$$

and

$$\begin{array}{ccc} \left|{\left(U_{1}^{\left(k\right)}y\right)}^{\prime }\left(s\right)\right| & \leq & \frac{1}{\Gamma (p)}∥R_{k}∥\sigma_{k}(|y_{k}\left(\varsigma \right)|+|D^{q}y_{k}\left(\varsigma \right)|+|y_{k}^{\prime }\left(\varsigma \right)|+|y_{k}^{\prime \prime }\left(\varsigma \right)|),\\ \left|{\left(U_{1}^{\left(k\right)}y\right)}^{\prime \prime }\left(s\right)\right| & \leq & \frac{1}{\Gamma (p-1)}∥R_{k}∥\sigma_{k}(|y_{k}\left(\varsigma \right)|+|D^{q}y_{k}\left(\varsigma \right)|+|y_{k}^{\prime }\left(\varsigma \right)|+|y_{k}^{\prime \prime }\left(\varsigma \right)|), \end{array}$$

for all *y*∈ $O_{\varpi_{k}}.$ Thus,

$$\begin{array}{ccc} {∥U_{1}y∥}_{B} & = & \sum_{k=1}^{25}{∥U_{1}^{\left(k\right)}y∥}_{\tilde{B}}\\ & \leq & \{\frac{p^{2}+1}{\Gamma (p+1)}+\frac{1}{\Gamma (p-q+1)}\}\sum_{k=1}^{25}∥R_{k}∥\sigma_{k}({∥y_{k}∥}_{\tilde{B}}). \end{array}$$

It shows that the operator $U_{1}$ is uniformly bounded on $O_{\varpi_{k}}$. Here, we need to prove the compactness of $U_{1}$ on $O_{\varpi_{k}}$. For this, let *s*_1_, *s*_2_ ∈ [0, 1] with *s*_1_ < *s*_2_, we have

$$\begin{array}{ccc} & & \left|(U_{1}^{\left(k\right)}y)\left(s_{2}\right)-(U_{1}^{\left(k\right)}y)\left(s_{1}\right)\right|\\ & \leq & |\int_{0}^{s_{2}}\frac{{\left(s_{2}-\varsigma \right)}^{p-1}}{\Gamma (p)}Z_{k}(\varsigma ,y_{k}\left(\varsigma \right),D^{q}y_{k}\left(\varsigma \right),y_{k}^{\prime }\left(\varsigma \right),y_{k}^{\prime \prime }\left(\varsigma \right))d\varsigma \\ & & -\int_{0}^{s_{1}}\frac{{\left(s_{1}-\varsigma \right)}^{p-1}}{\Gamma (p)}Z_{k}(\varsigma ,y_{k}\left(\varsigma \right),D^{q}y_{k}\left(\varsigma \right),y_{k}^{\prime }\left(\varsigma \right),y_{k}^{\prime \prime }\left(\varsigma \right))d\varsigma |\\ & \leq & \left|\int_{0}^{s_{1}}\frac{{\left(s_{2}-\varsigma \right)}^{p-1}-{\left(s_{1}-\varsigma \right)}^{p-1}}{\Gamma (p)}Z_{k}(\varsigma ,y_{k}\left(\varsigma \right),D^{q}y_{k}\left(\varsigma \right),y_{k}^{\prime }\left(\varsigma \right),y_{k}^{\prime \prime }\left(\varsigma \right))d\varsigma \right|\\ & & +|\int_{s_{1}}^{s_{2}}\frac{{\left(s_{2}-\varsigma \right)}^{p-1}}{\Gamma (p)}Z_{k}(\varsigma ,y_{k}\left(\varsigma \right),D^{q}y_{k}\left(\varsigma \right),y_{k}^{\prime }\left(\varsigma \right),y_{k}^{\prime \prime }\left(\varsigma \right))d\varsigma |\\ & \leq & \int_{0}^{s_{1}}\frac{{\left(s_{2}-\varsigma \right)}^{p-1}-{\left(s_{1}-\varsigma \right)}^{p-1}}{\Gamma (p)}|Z_{k}(\varsigma ,y_{k}\left(\varsigma \right),D^{q}y_{k}\left(\varsigma \right),y_{k}^{\prime }\left(\varsigma \right),y_{k}^{\prime \prime }\left(\varsigma \right))|d\varsigma \\ & & +\int_{s_{1}}^{s_{2}}\frac{{\left(s_{2}-\varsigma \right)}^{p-1}}{\Gamma (p)}|Z_{k}(\varsigma ,y_{k}\left(\varsigma \right),D^{q}y_{k}\left(\varsigma \right),y_{k}^{\prime }\left(\varsigma \right),y_{k}^{\prime \prime }\left(\varsigma \right))|d\varsigma \\ & \leq & \{\frac{s_{2}^{p}-s_{1}^{p}-{\left(s_{2}-s_{1}\right)}^{p}}{\Gamma (p+1)}+\frac{{\left(s_{2}-s_{1}\right)}^{p}}{\Gamma (p+1)}\}∥R_{k}∥\sigma_{k}({∥y_{k}∥}_{\tilde{B}}). \end{array}$$

Hence, $|(U_{1}^{\left(k\right)}y)\left(s_{2}\right)-(U_{1}^{\left(k\right)}y)\left(s_{1}\right)|\to 0$ as *s*_1_ → *s*_2_. Also, we have

$$\begin{array}{c} \underset{s_{1}\to s_{2}}{\text{lim}}|(D^{q}U_{1}^{\left(k\right)}y)\left(s_{2}\right)-(D^{q}U_{1}^{\left(k\right)}y)\left(s_{1}\right)|=0, \end{array}$$

and

$$\begin{array}{c} \underset{s_{1}\to s_{2}}{\text{lim}}|{\left(U_{1}^{\left(k\right)}y\right)}^{\prime }\left(s_{2}\right)-{\left(U_{1}^{\left(k\right)}y\right)}^{\prime }\left(s_{1}\right)|=0,\;\underset{s_{1}\to s_{2}}{\text{lim}}|{\left(U_{1}^{\left(k\right)}y\right)}^{\prime \prime }\left(s_{2}\right)-{\left(U_{1}^{\left(k\right)}y\right)}^{\prime \prime }\left(s_{1}\right)|=0. \end{array}$$

Hence, we deduce that the operators $U_{k}\;\left(k=1,2,\ldots ,25\right)$ are equicontinuous, which implies that U is equicontinuous. The Arzela-Ascoli theorem indeed reveals the compactness of the operator $U_{1}$ on $O_{\varpi_{k}}$.

Lastly, we need to prove that $U_{2}$ is a contraction mapping. For this, let $y,z\in O_{\varpi_{k}}.$ Thus, we have

$$\begin{array}{ccc} & & \left|(U_{2}^{\left(k\right)}z)\left(s\right)-(U_{2}^{\left(k\right)}y)\left(s\right)\right|\\ & \leq & \frac{|\mu_{3}|}{|\mu_{3}-\mu_{1}|}\int_{0}^{1}\int_{0}^{\varsigma }\frac{{\left(\varsigma -\tau \right)}^{p-1}}{\Gamma (p)}K_{k}\left(\tau \right)(|z_{k}\left(\tau \right)-y_{k}\left(\tau \right)|+|D^{q}z_{k}\left(\tau \right)-D^{q}y_{k}\left(\tau \right)|).\\ & & +|z_{k}^{\prime }\left(\tau \right)-y_{k}^{\prime }\left(\tau \right)|+|z_{k}^{\prime \prime }\left(\tau \right)-y_{k}^{\prime \prime }\left(\tau \right)|)d\tau d\varsigma +\frac{\Gamma \left(2-m\right)|\mu_{3}-2t\left(\mu_{3}-\mu_{1}\right)|}{|2\left(\mu_{3}-\mu_{1}\right)\left(\mu_{2}+\mu_{1}\Gamma \left(2-m\right)\right)|}\\ & & \left[\frac{|\mu_{1}|}{\Gamma (p)}\int_{0}^{1}{\left(1-\varsigma \right)}^{p-1}K_{k}\left(\varsigma \right)(|z_{k}\left(\varsigma \right)-y_{k}\left(\varsigma \right)|+|D^{q}z_{k}\left(\varsigma \right)-D^{q}y_{k}\left(\varsigma \right)|)+|z_{k}^{\prime }\left(\varsigma \right)-y_{k}^{\prime }\left(\varsigma \right)\right|\\ & & +|z_{k}^{\prime \prime }\left(\varsigma \right)-y_{k}^{\prime \prime }\left(\varsigma \right)|)d\varsigma +\frac{|\mu_{2}|}{\Gamma (p-m)}\int_{0}^{1}{\left(1-\varsigma \right)}^{p-m-1}K_{k}\left(\varsigma \right)(|z_{k}\left(\varsigma \right)-y_{k}\left(\varsigma \right)|\\ & & +|D^{q}z_{k}\left(\varsigma \right)-D^{q}y_{k}\left(\varsigma \right)|)+|z_{k}^{\prime }\left(\varsigma \right)-y_{k}^{\prime }\left(\varsigma \right)|+|z_{k}^{\prime \prime }\left(\varsigma \right)-y_{k}^{\prime \prime }\left(\varsigma \right)|)d\varsigma ]\\ & \leq & ∥K_{k}∥L_{0}^{*}{∥z_{k}-y_{k}∥}_{\tilde{B}} \end{array}$$

for each *k* = 1, 2, …, 25, where $L_{0}^{*}$ is given in (3.7). Also, by the similar computations, we have

$$\begin{array}{c} \underset{s\in [0,1]}{\text{sup}}|(D^{q}U_{2}^{\left(k\right)}z)\left(s\right)-(D^{q}U_{2}^{\left(k\right)}y)\left(s\right)|\leq ∥K_{k}∥L_{1}^{*}{∥z_{k}-y_{k}∥}_{\tilde{B}}. \end{array}$$

$$\begin{array}{c} \underset{s\in [0,1]}{\text{sup}}|{\left(U_{2}^{\left(k\right)}z\right)}^{\prime }\left(s\right)-{\left(U_{2}^{\left(k\right)}y\right)}^{\prime }\left(s\right)|\leq ∥K_{k}∥L_{2}^{*}{∥z_{k}-y_{k}∥}_{\tilde{B}}, \end{array}$$

$$\begin{array}{c} \underset{s\in [0,1]}{\text{sup}}|{\left(U_{2}^{\left(k\right)}z\right)}^{\prime \prime }\left(s\right)-{\left(U_{2}^{\left(k\right)}y\right)}^{\prime \prime }\left(s\right)|\leq 0. \end{array}$$

where $L_{1}^{*}$ and $L_{2}^{*}$ are given in (3.8) and (3.9), respectively. Thus, we have

$$\begin{array}{ccc} {∥U_{2}z-U_{2}y∥}_{B} & = & \sum_{k=1}^{25}{∥U_{2}^{\left(k\right)}z-U_{2}^{\left(k\right)}y∥}_{\tilde{B}}\\ & \leq & (L_{0}^{*}+L_{1}^{*}+L_{2}^{*})\sum_{k=1}^{25}∥K_{k}∥{∥z_{k}-y_{k}∥}_{\tilde{B}}, \end{array}$$

and so

$$\begin{array}{c} {∥U_{2}z-U_{2}y∥}_{B}\leq M{∥z-y∥}_{B}. \end{array}$$

As M<1, which means that $U_{2}$ is a contraction on $O_{\varpi_{k}}.$ We deduce that U possesses a fixed point that is a solution to the fractional boundary value problem (1.1) as a consequence of Theorem 2.3.

## 4 Examples

In this section, we present the following two examples to illustrate the relevance of our key findings.

**Example 4.1** Consider the system of differential equations given below

$$\begin{array}{c} \left\{ \begin{array}{cc} D^{1.5}y_{1}\left(s\right) & =\frac{e^{s}\;\text{arctan}\;y_{1}\left(s\right)}{5000}+0.0002e^{s}\;\text{sin}(D^{0.08}y_{1}\left(s\right))+\frac{30e^{s}{\left[y_{1}^{\prime }\left(s\right)\right]}^{2}}{150000(1+{\left[y_{1}^{\prime }\left(s\right)\right]}^{2})}\\ & \;+\frac{e^{s}\;{\text{sinh}}^{-1\;}y_{1}^{\prime \prime }\left(s\right)}{5000},\\ D^{1.5}y_{2}\left(s\right) & =0.008s\;\text{sin}y_{2}\left(s\right)+\frac{160s{\left[D^{0.08}y_{2}\left(s\right)\right]}^{2}}{2000+2000{\left[D^{0.08}y_{2}\left(s\right)\right]}^{2}}+\frac{8s\;{\text{sinh}}^{-1\;}y_{2}^{\prime }\left(s\right)}{1000}\\ & \;+\frac{80s\;\text{sin}y_{2}^{\prime \prime }\left(s\right)}{1000},\\ D^{1.5}y_{3}\left(s\right) & =\frac{e^{s}{\left[y_{3}\left(s\right)\right]}^{2}}{2500(1+{\left[y_{3}\left(s\right)\right]}^{2})}+0.0004e^{s}\;\text{sin}(D^{0.08}y_{3}\left(s\right))+\frac{2e^{s}\;{\text{sinh}}^{-1\;}y_{3}^{\prime }\left(s\right)}{5000}\\ & \;+\frac{8e^{s}\;\text{arctan}\;y_{3}^{\prime \prime }\left(s\right)}{20000}, \end{array}\right. \end{array}$$ (4.1)

with boundary conditions

$$\begin{array}{c} \left\{ \begin{array}{cc} 2y_{1}\left(0\right)+8(D^{0.08}y_{1}\left(0\right)) & =10\int_{0}^{1}y_{1}\left(\varsigma \right)d\varsigma \\ 2y_{1}\left(1\right)+8(D^{0.08}y_{1}\left(1\right)) & =10\int_{0}^{1}y_{1}\left(\varsigma \right)d\varsigma \\ 2y_{2}\left(0\right)+8(D^{0.08}y_{2}\left(0\right)) & =10\int_{0}^{1}y_{2}\left(\varsigma \right)d\varsigma \\ 2y_{2}\left(1\right)+8(D^{0.08}y_{2}\left(1\right)) & =10\int_{0}^{1}y_{2}\left(\varsigma \right)d\varsigma \\ 2y_{3}\left(0\right)+8(D^{0.08}y_{3}\left(0\right)) & =10\int_{0}^{1}y_{3}\left(\varsigma \right)d\varsigma \\ 2y_{3}\left(1\right)+8(D^{0.08}y_{3}\left(1\right)) & =10\int_{0}^{1}y_{3}\left(\varsigma \right)d\varsigma \end{array}\right. \end{array}$$ (4.2)

where *p* = 1.5, *q* = 0.08, *μ*_1_ = 2, *μ*_2_ = 8 and *μ*_3_ = 10.

Let $Z_{1},Z_{2},Z_{3}:\left[0,1\right]\times R\times R\times R\times R\to R$ are continuous functions given by

$$\begin{array}{c} \left\{ \begin{array}{cc} Z_{1}(s,z_{1},z_{2},z_{3},z_{4}) & =\frac{e^{s}\;\text{arctan}\;z_{1}}{5000}+0.0002e^{s}\;\text{sin}\;z_{2}\\ & \;+\frac{30e^{s}{\left[z_{3}^{\prime }\right]}^{2}}{150000(1+{\left[z_{3}^{\prime }\right]}^{2})}+\frac{e^{s}\;{\text{sinh}}^{-1\;}z_{4}^{\prime \prime }}{5000},\\ Z_{2}(s,z_{1},z_{2},z_{3},z_{4}) & =0.008s\;\text{sin}\;z_{1}+\frac{160s{\left[z_{2}\right]}^{2}}{2000+2000{\left[z_{2}\right]}^{2}}\\ & \;+\frac{8s\;{\text{sinh}}^{-1\;}z_{3}^{\prime }}{1000}+\frac{80s\;\text{sin}\;z_{4}^{\prime \prime }}{1000},\\ Z_{3}(s,z_{1},z_{2},z_{3},z_{4}) & =\frac{e^{s}{\left[z_{1}\right]}^{2}}{2500(1+{\left[z_{1}\right]}^{2})}+0.0004e^{s}\;\text{sin}\;z_{2}\\ & \;+\frac{2e^{s}\;{\text{sinh}}^{-1\;}z_{3}^{\prime }}{5000}+\frac{8e^{s}\;\text{arctan}\;z_{4}^{\prime \prime }}{20000}, \end{array}\right. \end{array}$$

for all *s* ∈ [0, 1], $z_{1},z_{2},z_{3},z_{4}\in R,$ whereas $Z_{4},Z_{5},\ldots ,Z_{25}:\left[0,1\right]\times R\times R\times R\times R\to R$ are zero functions. For each *s* ∈ [0, 1], $z_{1}^{⋆},z_{2}^{⋆},z_{3}^{⋆},z_{4}^{⋆},z_{1},z_{2},z_{3},z_{4}\in R,$ we have

$$\begin{array}{ccc} & & \left|Z_{1}(s,z_{1}^{⋆},z_{2}^{⋆},z_{3}^{⋆},z_{4}^{⋆})-Z_{1}(s,z_{1},z_{2},z_{3},z_{4})\right|\\ & \leq & \frac{e^{s}}{5000}(|\text{arctan}\;z_{1}^{⋆}-\text{arctan}\;z_{1}|+|\text{sin}\;z_{2}^{⋆}-\text{sin}\;z_{2}|+|\frac{{\left[z_{3}^{\prime ⋆}\right]}^{2}}{1+{\left[z_{3}^{\prime ⋆}\right]}^{2}}-\frac{{\left[z_{3}^{\prime }\right]}^{2}}{1+{\left[z_{3}^{\prime }\right]}^{2}}|\\ & & +|\;{\text{sinh}}^{-1\;}z_{4}^{⋆}\;-\;{\text{sinh}}^{-1\;}z_{4}|)\\ & \leq & \frac{e^{s}}{5000}(|z_{1}^{⋆}-z_{1}|+|z_{2}^{⋆}-z_{2}|+|z_{3}^{⋆}-z_{3}|+|z_{4}^{⋆}-z_{4}|),\\ & & \left|Z_{2}(s,z_{1}^{⋆},z_{2}^{⋆},z_{3}^{⋆},z_{4}^{⋆})-Z_{2}(s,z_{1},z_{2},z_{3},z_{4})\right|\\ & \leq & \frac{8s}{1000}(|\text{sin}\;z_{1}^{⋆}-\text{sin}\;z_{1}|+|\frac{{\left[z_{2}^{⋆}\right]}^{2}}{1+{\left[z_{2}^{⋆}\right]}^{2}}-\frac{{\left[z_{2}\right]}^{2}}{1+{\left[z_{2}\right]}^{2}}|+|\;{\text{sinh}}^{-1\;}z_{3}^{⋆}\left(s\right)-\;{\text{sinh}}^{-1\;}z_{3}\left(s\right)|\\ & & +|\text{sin}\;z_{4}^{⋆}-\text{sin}\;z_{4}|)\\ & \leq & \frac{8s}{1000}(|z_{1}^{⋆}-z_{1}|+|z_{2}^{⋆}-z_{2}|+|z_{3}^{⋆}-z_{3}|+|z_{4}^{⋆}-z_{4}|),\\ & & \left|Z_{3}(s,z_{1}^{⋆},z_{2}^{⋆},z_{3}^{⋆},z_{4}^{⋆})-Z_{3}(s,z_{1},z_{2},z_{3},z_{4})\right|\\ & \leq & \frac{e^{s}}{2500}(|\frac{{\left[z_{1}^{⋆}\right]}^{2}}{1+{\left[z_{1}^{⋆}\right]}^{2}}-\frac{{\left[z_{1}\right]}^{2}}{1+{\left[z_{1}\right]}^{2}}|+|\text{sin}\;z_{2}^{⋆}-\text{sin}\;z_{2}|+|\;{\text{sinh}}^{-1\;}z_{3}^{⋆}-\;{\text{sinh}}^{-1\;}z_{3}|\\ & & +|\text{arctan}\;z_{4}^{⋆}-\text{arctan}\;z_{4}|)\\ & \leq & \frac{e^{s}}{2500}(|z_{1}^{⋆}-z_{1}|+|z_{2}^{⋆}-z_{2}|+|z_{3}^{⋆}-z_{3}|+|z_{4}^{⋆}-z_{4}|). \end{array}$$

Here, $K_{1}\left(s\right)=\frac{e^{s}}{5000},K_{2}\left(s\right)=\frac{8s}{1000},K_{3}\left(s\right)=\frac{e^{s}}{2500},$ and $K_{4}\left(s\right)=K_{5}\left(s\right)=\cdots =K_{25}\left(s\right)=0,$ where $∥K_{1}∥=\frac{1}{5000},∥K_{2}∥=\frac{8}{1000},∥K_{3}∥=\frac{1}{2500},$ and $∥K_{4}∥=∥K_{5}∥=\cdots =∥K_{25}∥=0$. Let $\sigma_{1},\sigma_{2},\ldots ,\sigma_{25}:\left[0,\infty \right)\to R$ are identity functions. Then we obtain

$$\begin{array}{ccc} \left|Z_{1}\left(s,z_{1},z_{2},z_{3},z_{4}\right)\right| & \leq & \frac{e^{s}}{5000}(|\text{arctan}\;z_{1}|+|\text{sin}\;z_{2}|+|\frac{{\left[z_{3}^{\prime }\right]}^{2}}{1+{\left[z_{3}^{\prime }\right]}^{2}}|+|\;{\text{sinh}}^{-1\;}z_{4}|)\\ & \leq & \frac{e^{s}}{5000}(|z_{1}|+|z_{2}|+|z_{3}|+|z_{4}|) \end{array}$$

for all *s* ∈ [0, 1], $z_{1},z_{2},z_{3},z_{4}\in R.$ Also,

$$\begin{array}{ccc} \left|Z_{2}\left(s,z_{1},z_{2},z_{3},z_{4}\right)\right| & \leq & \frac{8s}{1000}(|\text{sin}\;z_{1}|+|\frac{{\left[z_{2}\right]}^{2}}{1+{\left[z_{2}\right]}^{2}}|+|\;{\text{sinh}}^{-1\;}z_{3}|+|\text{sin}\;z_{4}|)\\ & \leq & \frac{8s}{1000}(|z_{1}|+|z_{2}|+|z_{3}|+|z_{4}|) \end{array}$$

and

$$\begin{array}{ccc} \left|Z_{3}\left(s,z_{1},z_{2},z_{3},z_{4}\right)\right| & \leq & \frac{e^{s}}{2500}(|\frac{{\left[z_{1}\right]}^{2}}{1+{\left[z_{1}\right]}^{2}}|+|\text{sin}\;z_{2}|+|\;{\text{sinh}}^{-1\;}z_{3}|+|\text{arctan}\;z_{4}|)\\ & \leq & \frac{e^{s}}{2500}(|z_{1}|+|z_{2}|+|z_{3}|+|z_{4}|), \end{array}$$

for all *s* ∈ [0, 1], $z_{1},z_{2},z_{3},z_{4}\in R.$

Moreover, the continuous functions $R_{1},R_{2},\ldots ,R_{25}:\left[0,1\right]\to R$ are defined by

$$\begin{array}{c} R_{1}\left(s\right)=\frac{e^{s}}{5000},\;R_{2}\left(s\right)=\frac{8s}{1000},\;R_{3}\left(s\right)=\frac{e^{s}}{2500},\;and\;R_{4}\left(s\right)=R_{5}\left(s\right)=\cdots =R_{25}\left(s\right)=0. \end{array}$$

Also,

$$\begin{array}{c} L_{0}^{*}≃0.6741,\;L_{1}^{*}≃0.8155\;\text{and}\;L_{2}^{*}≃0.7902, \end{array}$$

and so

$$\begin{array}{c} L_{0}^{*}+L_{1}^{*}+L_{2}^{*}≃2.2798. \end{array}$$

Hence

$$\begin{array}{c} M≔(L_{0}^{*}+L_{1}^{*}+L_{2}^{*})(∥K_{1}∥+∥K_{2}∥+∥K_{3}∥)≃0.0219<1. \end{array}$$

It can be seen that all the conditions of Theorem 3.2 are satisfied, therefore, the proposed problem (4.1)–(4.2) has a solution.

**Example 4.2** Consider the system of fractional differential equations given below

$$\begin{array}{c} \left\{ \begin{array}{cc} D^{1.75}y_{1}\left(s\right) & =\frac{16s}{1000}{\text{sinh}}^{-1\;}y_{1}\left(s\right)+\frac{48{\left[D^{0.3}y_{1}\left(s\right)\right]}^{2}s}{3000+3000{\left[D^{0.3}y_{1}\left(s\right)\right]}^{2}}+0.016s\;{\text{sinh}}^{-1\;}y_{1}^{\prime }\left(s\right)\\ & \;+\frac{64s{\left[\text{sin}\;y_{1}^{\prime \prime }\left(s\right)\right]}^{2}}{4000(1+{\left[\text{sin}\;y_{1}^{\prime \prime }\left(s\right)\right]}^{2})}\\ D^{1.75}y_{2}\left(s\right) & =\frac{51{\left[\text{sin}\;y_{2}\left(s\right)\right]}^{2}e^{s}}{9000(1+{\left[\text{sin}\;y_{2}\left(s\right)\right]}^{2})}+\frac{17e^{s}}{3000}\text{sin}\;(D^{0.3}y_{2}\left(s\right))\\ & \;+\frac{34{\left[\text{arctan}\;y_{2}^{\prime }\left(s\right)\right]}^{2}e^{s}}{6000+6000{\left[\text{arctan}\;y_{2}^{\prime }\left(s\right)\right]}^{2}}+\frac{85e^{s}}{15000}\;{\text{sinh}}^{-1\;}y_{2}^{\prime \prime }\left(s\right)\\ D^{1.75}y_{3}\left(s\right) & =0.0016s\;\text{arctan}\;y_{3}\left(s\right)+\frac{32s{\left[D^{0.3}y_{3}\left(s\right)\right]}^{2}}{20000(1+{\left[D^{0.3}y_{3}\left(s\right)\right]}^{2})}+\frac{8s}{5000}\;{\text{sinh}}^{-1\;}y_{3}^{\prime }\left(s\right)\\ & \;+\frac{16s{\left[\text{sin}\;y_{3}^{\prime \prime }\left(s\right)\right]}^{2}}{10000+10000{\left[\text{sin}\;y_{3}^{\prime \prime }\left(s\right)\right]}^{2}} \end{array}\right. \end{array}$$ (4.3)

with boundary conditions

$$\begin{array}{c} \left\{ \begin{array}{cc} \frac{3}{11}y_{1}\left(0\right)+\frac{17}{38}(D^{0.3}y_{1}\left(0\right)) & =\frac{11}{51}\int_{0}^{1}y_{1}\left(\varsigma \right)d\varsigma \\ \frac{3}{11}y_{1}\left(1\right)+\frac{17}{38}(D^{0.3}y_{1}\left(1\right)) & =\frac{11}{51}\int_{0}^{1}y_{1}\left(\varsigma \right)d\varsigma \\ \frac{3}{11}y_{2}\left(0\right)+\frac{17}{38}(D^{0.3}y_{2}\left(0\right)) & =\frac{11}{51}\int_{0}^{1}y_{2}\left(\varsigma \right)d\varsigma \\ \frac{3}{11}y_{2}\left(1\right)+\frac{17}{38}(D^{0.3}y_{2}\left(1\right)) & =\frac{11}{51}\int_{0}^{1}y_{2}\left(\varsigma \right)d\varsigma \end{array}\right. \end{array}$$

$$\begin{array}{c} \left\{ \begin{array}{cc} \frac{3}{11}y_{3}\left(0\right)+\frac{17}{38}(D^{0.3}y_{3}\left(0\right)) & =\frac{11}{51}\int_{0}^{1}y_{3}\left(\varsigma \right)d\varsigma \\ \frac{3}{11}y_{3}\left(1\right)+\frac{17}{38}(D^{0.3}y_{3}\left(1\right)) & =\frac{11}{51}\int_{0}^{1}y_{3}\left(\varsigma \right)d\varsigma \end{array}\right. \end{array}$$ (4.4)

where $p=1.75,q=0.3,\mu_{1}=\frac{3}{11},\mu_{2}=\frac{17}{38}$ and $\mu_{3}=\frac{11}{51}$. Let $Z_{1},Z_{2},Z_{3}:\left[0,1\right]\times R\times R\times R\times R\to R$ are continuous functions given by

$$\begin{array}{c} \left\{ \begin{array}{cc} Z_{1}(s,z_{1},z_{2},z_{3},z_{4}) & =\frac{16s}{1000}\;{\text{sinh}}^{-1\;}z_{1}+\frac{48{\left[z_{2}\right]}^{2}s}{3000+3000{\left[z_{2}\right]}^{2}}\\ & \;+0.016s\;{\text{sinh}}^{-1\;}z_{3}+\frac{64s{\left[\text{sin}\;z_{4}\right]}^{2}}{4000(1+{\left[\text{sin}\;z_{4}\right]}^{2})},\\ Z_{2}(s,z_{1},z_{2},z_{3},z_{4}) & =\frac{51e^{s}{\left[\text{sin}\;z_{1}\right]}^{2}}{9000(1+{\left[\text{sin}\;z_{1}\right]}^{2})}+\frac{17e^{s}}{3000}\text{sin}\;z_{2}\\ & \;+\frac{34{\left[\text{arctan}\;z_{3}\right]}^{2}e^{s}}{6000+6000{\left[\text{arctan}\;z_{3}\right]}^{2}}+\frac{85e^{s}}{15000}\;{\text{sinh}}^{-1\;}z_{4},\\ Z_{3}(s,z_{1},z_{2},z_{3},z_{4}) & =0.0016s\;\text{arctan}\;z_{1}+\frac{32s{\left[z_{2}\right]}^{2}}{20000(1+{\left[z_{2}\right]}^{2})}\\ & \;+\frac{8s}{5000}\;{\text{sinh}}^{-1\;}z_{3}+\frac{16{\left[\text{sin}\;z_{4}\right]}^{2}s}{10000+10000{\left[\text{sin}\;z_{4}\right]}^{2}}, \end{array}\right. \end{array}$$

for all *s* ∈ [0, 1], $z_{1},z_{2},z_{3},z_{4}\in R,$ whereas $Z_{4},Z_{5},\ldots ,Z_{25}:\left[0,1\right]\times R\times R\times R\times R\to R$ are zero functions. For each *s* ∈ [0, 1], $z_{1}^{⋆},z_{2}^{⋆},z_{3}^{⋆},z_{4}^{⋆},z_{1},z_{2},z_{3},z_{4}\in R,$ we have

$$\begin{array}{ccc} & & \left|Z_{1}(s,z_{1}^{⋆},z_{2}^{⋆},z_{3}^{⋆},z_{4}^{⋆})-Z_{1}(s,z_{1},z_{2},z_{3},z_{4})\right|\\ & \leq & \frac{16s}{1000}(|\;{\text{sinh}}^{-1\;}z_{1}^{⋆}-\;{\text{sinh}}^{-1\;}z_{1}|+|\frac{{\left[z_{2}^{⋆}\right]}^{2}}{1+{\left[z_{2}^{⋆}\right]}^{2}}-\frac{{\left[z_{2}\right]}^{2}}{1+{\left[z_{2}\right]}^{2}}|\\ & & +|\;{\text{sinh}}^{-1\;}z_{3}^{⋆}-\;{\text{sinh}}^{-1\;}z_{3}|+|\frac{{\left[\text{sin}\;z_{4}^{⋆}\right]}^{2}}{1+{\left[\text{sin}\;z_{4}^{⋆}\right]}^{2}}-\frac{{\left[\text{sin}\;z_{4}\right]}^{2}}{1+{\left[\text{sin}\;z_{4}\right]}^{2}}|)\\ & \leq & \frac{16s}{1000}(|z_{1}^{⋆}-z_{1}|+|z_{2}^{⋆}-z_{2}|+|z_{3}^{⋆}-z_{3}|+|z_{4}^{⋆}-z_{4}|),\\ & & \left|Z_{2}(s,z_{1}^{⋆},z_{2}^{⋆},z_{3}^{⋆},z_{4}^{⋆})-Z_{2}(s,z_{1},z_{2},z_{3},z_{4})\right|\\ & \leq & \frac{17e^{s}}{3000}(|\frac{{\left[\text{sin}\;z_{1}^{⋆}\right]}^{2}}{1+{\left[\text{sin}\;z_{1}^{⋆}\right]}^{2}}-\frac{{\left[\text{sin}\;z_{1}\right]}^{2}}{1+{\left[\text{sin}\;z_{1}\right]}^{2}}|+|\text{sin}\;z_{2}^{⋆}-\text{sin}\;z_{2}|\\ & & +|\frac{{\left[\text{arctan}\;z_{3}^{⋆}\right]}^{2}}{1+{\left[\text{arctan}\;z_{3}^{⋆}\right]}^{2}}-\frac{{\left[\text{arctan}\;z_{3}\right]}^{2}}{1+{\left[\text{arctan}\;z_{3}\right]}^{2}}|+|\;{\text{sinh}}^{-1\;}z_{4}^{⋆}-\;{\text{sinh}}^{-1\;}z_{4}|)\\ & \leq & \frac{17e^{s}}{3000}(|z_{1}^{⋆}-z_{1}|+|z_{2}^{⋆}-z_{2}|+|z_{3}^{⋆}-z_{3}|+|z_{4}^{⋆}-z_{4}|),\\ & & \left|Z_{3}(s,z_{1}^{⋆},z_{2}^{⋆},z_{3}^{⋆},z_{4}^{⋆})-Z_{3}(s,z_{1},z_{2},z_{3},z_{4})\right|\\ & \leq & \frac{8s}{5000}(|\text{arctan}\;z_{1}^{⋆}-\text{arctan}\;z_{1}|+|\frac{{\left[z_{2}^{⋆}\right]}^{2}}{1+{\left[z_{2}^{⋆}\right]}^{2}}-\frac{{\left[z_{2}\right]}^{2}}{1+{\left[z_{2}\right]}^{2}}|\\ & & +|\;{\text{sinh}}^{-1\;}z_{3}^{⋆}-\;{\text{sinh}}^{-1\;}z_{3}|+|\frac{{\left[\text{sin}\;z_{4}^{⋆}\right]}^{2}}{1+{\left[\text{sin}\;z_{4}^{⋆}\right]}^{2}}-\frac{{\left[\text{sin}\;z_{4}\right]}^{2}}{1+{\left[\text{sin}\;z_{4}\right]}^{2}}|)\\ & \leq & \frac{8s}{5000}(|z_{1}^{⋆}-z_{1}|+|z_{2}^{⋆}-z_{2}|+|z_{3}^{⋆}-z_{3}|+|z_{4}^{⋆}-z_{4}|). \end{array}$$

Here, $K_{1}\left(s\right)=\frac{16s}{1000},K_{2}\left(s\right)=\frac{17e^{s}}{3000},K_{3}\left(s\right)=\frac{8s}{5000},$ and $K_{4}\left(s\right)=K_{5}\left(s\right)=\cdots =K_{25}\left(s\right)=0,$ where $∥K_{1}∥=\frac{16}{1000},∥K_{2}∥=\frac{17}{3000},∥K_{3}∥=\frac{8}{5000},$ and $∥K_{4}∥=∥K_{5}∥=\cdots =∥K_{25}∥=0$. Let $\sigma_{1},\sigma_{2},\ldots ,\sigma_{25}:\left[0,\infty \right)\to R$ be identity functions. Then we obtain

$$\begin{array}{ccc} \left|Z_{1}\left(s,z_{1},z_{2},z_{3},z_{4}\right)\right| & \leq & \frac{16s}{1000}(|\;{\text{sinh}}^{-1\;}z_{1}|+|\frac{{\left[z_{2}\right]}^{2}}{1+{\left[z_{2}\right]}^{2}}|+|\;{\text{sinh}}^{-1\;}z_{3}|+|\frac{{\left[\text{sin}\;z_{4}\right]}^{2}}{1+{\left[\text{sin}\;z_{4}\right]}^{2}}|)\\ & \leq & \frac{16s}{1000}(|z_{1}|+|z_{2}|+|z_{3}|+|z_{4}|). \end{array}$$

Also,

$$\begin{array}{ccc} \left|Z_{2}\left(s,z_{1},z_{2},z_{3},z_{4}\right)\right| & \leq & \frac{17e^{s}}{3000}(|\frac{{\left[\text{sin}\;z_{1}\right]}^{2}}{1+{\left[\text{sin}\;z_{1}\right]}^{2}}|+|\text{sin}\;z_{2}|+|\frac{{\left[\text{arctan}\;z_{3}\right]}^{2}}{1+{\left[\text{arctan}\;z_{3}\right]}^{2}}|+|\;{\text{sinh}}^{-1\;}z_{4}|)\\ & \leq & \frac{17e^{s}}{3000}(|z_{1}|+|z_{2}|+|z_{3}|+|z_{4}|) \end{array}$$

and

$$\begin{array}{ccc} \left|Z_{3}\left(s,z_{1},z_{2},z_{3},z_{4}\right)\right| & \leq & \frac{8s}{5000}(|\text{arctan}\;z_{1}|+|\frac{{\left[z_{2}\right]}^{2}}{1+{\left[z_{2}\right]}^{2}}|+|\;{\text{sinh}}^{-1\;}z_{3}|+|\frac{{\left[\text{sin}\;z_{4}\right]}^{2}}{1+{\left[\text{sin}\;z_{4}\right]}^{2}}|)\\ & \leq & \frac{8s}{5000}(|z_{1}|+|z_{2}|+|z_{3}|+|z_{4}|), \end{array}$$

for all *s* ∈ [0, 1], $z_{1},z_{2},z_{3},z_{4}\in R.$

Furthermore, the continuous functions $R_{1},R_{2},\ldots ,R_{25}:\left[0,1\right]\to R$ are defined by

$$\begin{array}{c} R_{1}\left(s\right)=\frac{16s}{1000},\;R_{2}\left(s\right)=\frac{17e^{s}}{5000},\;R_{3}\left(s\right)=\frac{8s}{5000},\;and\;R_{4}\left(s\right)=R_{5}\left(s\right)=\cdots =R_{25}\left(s\right)=0. \end{array}$$

Also,

$$\begin{array}{c} L_{0}^{*}≃1.803,\;L_{1}^{*}≃0.819\;\text{and}\;L_{2}^{*}≃0.745, \end{array}$$

and so

$$\begin{array}{c} L_{0}^{*}+L_{1}^{*}+L_{2}^{*}≃3.367. \end{array}$$

Hence

$$\begin{array}{c} M≔(L_{0}^{*}+L_{1}^{*}+L_{2}^{*})(∥K_{1}∥+∥K_{2}∥+∥K_{3}∥)≃0.077<1. \end{array}$$

It can be seen that all the conditions of Theorem 3.2 are fulfilled, hence, the boundary value problem (4.1)–(4.2) has a solution.

## 5 Conclusion and open problems

Chemical graph theory is a branch of mathematics in which graphs represent the molecular structures of chemical compounds, and specific mathematical challenges are studied using theoretical and analytical methodologies. In recent decades, the fast growth of this subject has resulted in the development of various ground-breaking and novel ideas and techniques for conducting such research. Several researchers have used the structure of star graphs to investigate the solutions of fractional differential equations. They used star graphs because their approach requires a center node with interconnections to nearby vertices but no node-to-node connections. Since, in general, the graphs can have several junction nodes, therefore in this article, we introduced the idea of an octane graph. We have analyzed a graph with vertices labeled by 0 or 1, which is inspired by a graph representation of the octane compound, and formulated fractional differential equations on each of its edges. The existence of solutions results to the suggested fractional differential equation have been investigated by utilizing the Krasnoselskii and Schaefer fixed point theorems. In the end, we presented two examples to demonstrate the importance of our findings.

Here, we give the following open problems for the interested readers.

Problem 1: Can we extend this idea to the circular ring type graphs?
Problem 2: Can we use another method that can guarantee the conclusion of the proposed results?

We also pose the stability of the proposed fractional differential equation (1.1) as an open problem.

## References

[1] Biggs NL. Algebraic Graph Theory. Cambridge: University Press; 1974.

[2] Cartwright D, Harary F. Structural balance: a generalization of Heider’s theory. Psychological Review. 1953; 63(5): 277–293. doi: 10.1037/h0046049 13359597

[3] Mattuck RD. A Guide to Feynman Diagrams in the Many-Body Problem. New York: McGrawHill; 1967.

[4] Avondo-Bodino G. Economic Applications of the Theory of Graphs. New York: Gordon & Breach; 1962.

[5] Harary F. Graph Theory and Theoretical Physics. New York: Academic Press; 1967. 10.1063/1.3035700

[6] Lane R. Elemente der Graphentheorie und ihre Anwendung in den biologischen Wissenschaften. Leipzig: Akademischer Verlag; 1970.

[7] Culik K. Application of Graph Theory to Mathematical Logic and Linguistics. Prague: Czechoslovak Academy of Sciences; 1964.

[8] Korach M, Hasko L. Acta Chem. Acad. Sci. Hung, 1972; 72, 77.

[9] Hage P, Harary F. Structural Models in Anthropology. London: Cambridge University Press; 1983.

[10] Flament C. Applications of Graph Theory to Group Structure. New Jersey: Prentice-Hall, Englewood Cliffs; 1963.

[11] Lissowski A. Theoretical consideration on movement of the myxomycete plasmoida. Dislocation and geometry of plasmoidal network on cylinders and cones. Acta Protozoologica. 1972; 11: 131–136.

[12] Roberts F. Applications of Combinatorics and Graph Theory to the Biological and Social Sciences. New York: Springer-Verlag; 1989.

[13] Johnson DE, Johnson JR. Graph Theory with Engineering Applications. New York: Ronald Press; 1972.

[14] Even S. Graph Algorithms. London: Pitman; 1979.

[15] Cliff A, Haggett P, Ord K. Graph Theory and Geography. In: Wilson RJ, Beineke LW, editors. Applications of Graph Theory. London: Academic Press; 1979. Ch. 10. [196, 2.3, 362].

[16] Rouvray DH. Graph theory in chemistry. Royal Institute of Chemistry Reviews. 1971; 4: 173–195. doi: 10.1039/rr9710400173

[17] Gutman I, Trinajstic N. Graph Theory and Molecular Orbitals. Topics Curr. Chem. 1973; 42: 49–93.

[18] Balaban AT. Chemical Applications of Graph Theory. London: Academic Press; 1976.

[19] Slanina Z. An interplay between the phenomenon of chemical isomerism and symmetry requirements: A perennial source of stimuli for molecular-structure concepts, as well as for algebraic and computational chemistry. Computers & Mathematics with Applications. 1986; 12: 585–616. doi: 10.1016/0898-1221(86)90413-X

[20] King RB, Rouvray DH. Chemical applications of topology and group theory. Theoret. Chim. Acta. 1986; 69: 1–10. doi: 10.1007/BF00526287

[21] King RB. Chemical Applications of Topology and Graph Theory. USA: Elsevier Science Publishers; 1983.

[22] Prelog V. Nobel Lecture. December 12, 1975. Reprinted in: Science. 1976; 193. 17.

[23] Lynch MJ, Harrison JM, Town VG, Ash JE. Computer Handling of Chemical Structure Information. London: Macdonald and Co; 1971.

[24] Carthart RE, Smith DH, Brown H, Djerassi C. Applications of artificial intelligence for chemical inference. XVII. Approach to computer-assisted elucidation of molecular structure. Journal of the American Chemical Society. 1975; 97(20): 5755–5762. doi: 10.1021/ja00853a021

[25] Trinajstic N, Nikolic S, Knop JV, Muller WR, Szymanski K. Computational Chemical Graph Theory: Characterization, Enumeration and Generation of Chemical Structures by Computer Methods. Chichester: Ellis Horwood Ltd; 1991.

[26] Corey EJ. Centenary lecture. Computer-assisted analysis of complex synthetic problems. Quarterly Reviews. Chemical Society. 1971; 25(4): 455–482. 10.1039/QR9712500455

[27] Hendrickson JB, Grier DL, Toczko AG. A logic-based program for synthesis design. J. Am. Chem. Soc. 1985; 107(18): 5228–5238. doi: 10.1021/ja00304a033

[28] Lumer G. Connecting of local operators and evolution equations on a network. Lect. Notes Math. 1985; 787:219–234. doi: 10.1007/BFb0086338

[29] Gordeziani DG, Kupreishvli M, Meladze HV, Davitashvili TD. On the solution of boundary value problem for differential equations given in graphs. Appl. Math. Lett. 2008; 13: 80–91.

[30] Zavgorodnii MG, Pokornyi YV. On the spectrum of second-order boundary value problems on spatial networks. Usp. Mat. Nauk. 1989; 44: 220–221.

[31] Graef JR, Kong LJ, Wang M. Existence and uniqueness of solutions for a fractional boundary value problem on a graph. Fract. Calc. Appl. Anal. 2014; 17: 499–510. doi: 10.2478/s13540-014-0182-4

[32] Mehandiratta V, Mehra M, Leugering G. Existence and uniqueness results for a nonlinear Caputo fractional boundary value problem on a star graph. J. Math. Anal. Appl. 2019; 477(2): 1243–1264. doi: 10.1016/j.jmaa.2019.05.011

[33] Turab A, Sintunavarat W. The novel existence results of solutions for a nonlinear fractional boundary value problem on the ethane graph. Alex. Eng. J. 2021; 60(6): 5365–5374. doi: 10.1016/j.aej.2021.04.020

[34] Turab A, Mitrović ZD, Savić A. Existence of solutions for a class of nonlinear boundary value problems on the hexasilinane graph. Adv Differ Equ. 2021; 494: 2021. 10.1186/s13662-021-03653-w

[35] Ali W, Turab A, Nieto JJ. On the novel existence results of solutions for a class of fractional boundary value problems on the cyclohexane graph. J Inequal Appl. 2022; 5: 2022.

[36] Etemad S, Rezapour S. On the existence of solutions for fractional boundary value problems on the ethane graph. Adv Differ Equ. 2020; 276: 2020. 10.1186/s13662-020-02736-4

[37] Rezapour S, Deressa CT, Hussain A, Etemad S, George R, Ahmad B. A theoretical analysis of a fractional multi-dimensional system of boundary value problems on the methylpropane graph via fixed point technique. Mathematics 2022; 10(4): 568. doi: 10.3390/math10040568

[38] Baleanu D, Etemad S, Mohammadi H, Rezapour S. A novel modeling of boundary value problems on the glucose graph. Communications in Nonlinear Science and Numerical Simulation 2021; 100: 1007–5704. doi: 10.1016/j.cnsns.2021.105844

[39] Shah K, Khan RA. Existence and Uniqueness Results to a Coupled System of Fractional Order Boundary Value Problems by Topological Degree Theory. Numerical Functional Analysis and Optimization 2016; 37(7): 887–899. doi: 10.1080/01630563.2016.1177547

[40] Shah K, Ali A, Khan RA. Degree theory and existence of positive solutions to coupled systems of multi-point boundary value problems. Bound Value Probl. 2016; 43: 2016. 10.1186/s13661-016-0553-3

[41] Wang J, Shah K, Ali A. Existence and Hyers-Ulam stability of fractional nonlinear impulsive switched coupled evolution equations. Math Meth Appl Sci. 2018; 41: 2392–2402.

[42] Turab A, Sintunavarat W. A unique solution of the iterative boundary value problem for a second-order differential equation approached by fixed point results. Alex. Eng. J. 2021; 60(6): 5797–5802. doi: 10.1016/j.aej.2021.04.031

[43] Baleanu D, Etemad S, Rezapour S. On a fractional hybrid integro-differential equation with mixed hybrid integral boundary value conditions by using three operators. Alexandria Engineering Journal 2020; 59(5): 3019–3027. doi: 10.1016/j.aej.2020.04.053

[44] Baleanu D, Etemad S, Rezapour S. A hybrid Caputo fractional modeling for thermostat with hybrid boundary value conditions. Bound Value Probl. 2020; 2020: 64. doi: 10.1186/s13661-020-01361-0

[45] Thabet ST, Etemad S, Rezapour S. On a coupled Caputo conformable system of pantograph problems. Turk. J. Math. 2021; 45(1): 496–519. doi: 10.3906/mat-2010-70

[46] Mohammadi H, Kumar S, Rezapour S, Etemad S. A theoretical study of the Caputo–Fabrizio fractional modeling for hearing loss due to Mumps virus with optimal control. Chaos, Solitons & Fractals 2021; 144. 10.1016/j.chaos.2021.110668

[47] Matar MM, Abbas MI, Alzabut J, Kaabar MKA, Etemad S, Rezapour S. Investigation of the p-Laplacian nonperiodic nonlinear boundary value problem via generalized Caputo fractional derivatives. Adv Differ Equ. 2021; 68: 2021. 10.1186/s13662-021-03228-9

[48] Alizadeh S, Baleanu D, Rezapour S. Analyzing transient response of the parallel RCL circuit by using the Caputo–Fabrizio fractional derivative. Adv Differ Equ. 2020; 55: 2020. 10.1186/s13662-020-2527-0 PMC7301114 32572336

[49] Baleanu D, Rezapour S, Saberpour Z. On fractional integro-differential inclusions via the extended fractional Caputo–Fabrizio derivation. Bound Value Probl. 2019; 79: 2019 10.1186/s13661-019-1194-0

[50] Baleanu D, Etemad S, Pourrazi S, Rezapour S. On the new fractional hybrid boundary value problems with three-point integral hybrid conditions. Adv Differ Equ. 2019; 473: 2019. 10.1186/s13662-019-2407-7

[51] Caputo M, Fabrizio M. A new definition of fractional derivative without singular kernel. Progress in Fractional Differentiation and Applications. 2015; 1(2): 1–13.

[52] Losada J, Nieto JJ. Properties of a new fractional derivative without singular kernel. Progress in Fractional Differentiation and Applications. 2015; 1(2): 87–92. 10.12785/pfda/010202

[53] Sintunavarat W, Turab A. Mathematical analysis of an extended SEIR model of COVID-19 using the ABC-fractional operator. Mathematics and Computers in Simulation 2022; 198: 65–84. doi: 10.1016/j.matcom.2022.02.009 35194306 PMC8851883

[54] Aydogan MS, Baleanu D, Mousalou A, Rezapour S. On high order fractional integro-differential equations including the Caputo–Fabrizio derivative. Bound Value Probl. 2018; 90: 2018. 10.1186/s13661-018-1008-9

[55] Baleanu D, Mohammadi H, Rezapour S. Analysis of the model of HIV-1 infection of *CD*4+ T-cell with a new approach of fractional derivative. Adv Differ Equ. 2020; 71: 2020. 10.1186/s13662-020-02544-w

[56] Smart DR. Fixed Point Theorems. Cambridge University Press; 1990.

